# Transformer-aided guard: A hybrid spatio-temporal framework for confidence-aware intrusion detection in cyber-physical systems

**DOI:** 10.1371/journal.pone.0361820

**Published:** 2026-11-04

**Authors:** Abeer Aljohani

**Affiliations:** Department of Computer Science and information, Applied College, Taibah University, Madinah, Saudi Arabia; King Abdulaziz University Faculty of Computing and Information Technology, SAUDI ARABIA

## Abstract

In this age of ubiquitous networks, Cyber-Physical Systems (CPSs) have evolved into an essentail part of critical infrastructures like smart grids and Internet of Things (IoT) ecosystems. The interconnectedness of cyber and physical components, however, makes CPSs more vulnerability to sophisticated and stealthy cyber intrusions which are typically untraceable by conventional Intrusion Detection Systems (IDS). Even though Graph Neural Networks (GNNs) are adopted with the aim of strengthening the performance of IDS, there are several challenges noted with available models including limited accuracy. Also, their capacity to capture the dynamic and structured dependencies within CPS environments is insufficient. Within the background, the present study proposes the Transformer-Aided Guard (TAG) approach. This is a novel hybrid spatio-temporal framework, designed for confidence-aware and precise intrusion detection. Through the Swin Transformer and spatial relationship learning using a Spatio-Temporal Graph Neural Network (STGNN), the network leverages temporal sequence modelling. Spatial and temporal embeddings are integrated by a dual-stage infusion mechanism, making it possible to attain more complex learning. Furthermore, a post-hoc Bayesian inference layer based on Monte Carlo Dropout (MC-Dropout) is incorporated to measure prediction uncertainty and enhance decision reliability. Extensive evaluations performed on the benchmark datasets revealed effectiveness of the framework as a promising average accuracy of 98% is achieved. Applicability of the framework is also validated through its detection latency across diverse CPS environments, whereas the ablation study conducted validate the contribution of each architectural component. The findings testify TAG as a secure, scalable, and reliable intrusion detection framework for the next-generation CPS applications.

## Introduction

The development of interconnected intelligent technologies has transformed industrial ecosystems. This equally increases the need for a smooth integration between communication networks and physical processes. Cyber-Physical Systems (CPS) constitute the technological backbone of such intelligent and autonomous infrastructures. With CPS, coordinated computational intelligence is incorporated with real-world physical operations in a unified way in real-time. In these systems, sensor networks are always collecting data, while actuators implement control commands on the basis of analytical feedback, establishing a powerful closed-loop architecture that facilitates independent decision making in fast-changing situations. The increasing convergence of cyber and physical components has significantly expanded the attack surface of CPS environments. They are becomming more exposed to stealthy and evolving cyber threats that often evade traditional IDSs. Most of such threats are both multistage and too challenging to identify using standard protection mechanisms [1]. Some of the attacks attemp to breach detection layers for unauthorized access while others try to intricate attack chains through initial compromise, privilege escalation, or data infiltration.

To address the growing security challenges of modern CPS, a wide range IDS have been developed. Despite considerable progress, the conventional IDS solutions remain inadequate for capturing the complex spatial dependencies and evolving temporal dynamics inherent in heterogeneous CPS environments. The standard signature-based and rule-based systems laregly depend on predefined knowledge and struggle to identify rapidly evolving threats [2]. To overcome the challenges, researchers have explored Machine Learning (ML)-based IDS frameworks, particularly in dynamic network environments [3,4]. Such systems leverage data-driven intelligence to automatically learn complex behavioural patterns and detect anomalous activities. However, ML-based solutions shallow contextual understanding which dimishes their ability to model the distributed and rapidly changing behaviour of CPS [5]. It is within this background that Graph Networks (GNNs) are merging as a promising alternative for context-aware intrusion detection. GNN-based IDS are able to identify complicated relational dependencies among entities that are networked. They are, therefore, able to support structured-aware and scalable monitoring [6]. Because they are able to represent both physical and cyber components as graph entities, it is possible for these models to extract local and physical behavioural patterns. Nonetheless, the GNN frameworks are computationally expensive. Besides, majority of GNN-based approaches are only able to provide static snapshots of CPS behaviour and neglect the temporal dynamics. Consequently, time-dependent or slow-evolving attacks–-such as multi-stage intrusions and stealthy probing may remain undetected. Despite significant advancements in IDS, securing CPS remains a challenging task because of the complex interplay of spatial, temporal, and relational dependencies of data. Though, recent studies have explored the integration of graph-based learning and sequential modelling, in most hybrid IDS the architectural choices are insufficiently articulated. Capturing the complementary dependencies; temporal and spatial, in isolation can lead to fragmented contextual representations. This limits a model’s ability to characterize complex attack behaviours. Moreover, the contemporary frameworks operate under deterministic assumptions without predictive uncertainty which limits their reliability in safety-critical CPS environments. These limitations highlight the need of a unified end-to-end framework that simultaneously captures structural and temporal dynamics with predictive uncertainty. A promising direction to overcome these limitations is the design of a robust and unified framework capable of effectively capturing spatio-temporal dependencies with reliable confidence-aware predictions across diverse CPS environments, hence this research.

With this study, a novel hybrid spatio-temporal framework named TAG is proposed for effective and reliable intrusion detection in CPS. The TAG architecture synergistically integrates the Swin Transformer [7] with the Spatio-Temporal Graph Neural Network (STGNN) [6]; thereby capturing both temporal evolution and spatial topology. In the Swin Transformer, the modelling of time-related behaviours occurs through shifted-window multi-head self-attention instruments. On the other hand, space-related dependencies among CPS entities are learned via STGNN. A dual-stage fusion strategy is then used to amalgamate the time-based and space-based embeddings into a unified presentation, making it possible to attain context-aware downstream intrusion classification. Further reliability is attained by post-hoc Bayesian inference based on Monte Carlo Dropout (MC-Dropout) in the inference pipeline. With the ability to perform numerous stochastic forward passes, the model quantifies predictive uncertainty and improve confidence-aware decision-making. Differing from previous works that overlooked time-based and graph-isomorphism, TAG efficiently captures long-sequence behaviours through a window-based self-attention design. Moreover, where the fusion strategy of contemporary approaches rely on feature concatenation, TAG employs attention-based fusion to adaptively integrate heterogeneous features. Four diverse benchmark datasets are used for training and cross-domain generalisation. Two of the datasets are node-level; SWaT and N-BaIoT whereas two are graph-level (network-flow) datasets; UNSW-NB15 and TON-IoT. The terms node-level and graph-level describe the granularity at which the corresponding benchmark data are represented and processed by the learning framework. Node-level representations are about attack states with individual CPS entities, whereas graph-level representations model interactions among multiple entities. As per the evaluation results obtained, an average True Positive Rate (TPR) of 0.96 and an average F1-score of 0.98 is achieved highlighting effectiveness of TAG across diverse CPS environments. Furthermore, Bayesian uncertainty analysis shows that most predictions fall outside the uncertainty zone, confirming the reliability and robustness of TAG. A competitive detection throughput of 9.7 attacks per second is also achieved by the framework. The evaluation results indicate applicability of the proposed framework across large-scale CPS infrastructure. Main contributions of the study are summarised below:

- We propose a novel spatio-temporal architecture that integrates adaptive graph-based spatial representation with transformer-driven temporal modelling so that to ensure wide-ranging modelling of CPS behaviour.
- We introduce a cross-attention-base dual-fusion embeddings mechanism to support adaptive amalgamation of fixed features and higher-level spatio-temporal representations.
- We incorporate post-hoc Bayesian inference through MC-Dropout to quantify prediction confidence and ensure accurate decision-making in safety-critical systems.

Wide-ranging evaluation across four datasets validated the promising accuracy, stability, and execution speed of the proposed system. The experimentation reults highlight potential of TAG for safeguarding next-generation cyber-physical infrastructures against the sophisticated cyber threats. Overall architecture of the proposed framework is illustrated in Fig 1.

**Fig 1:**
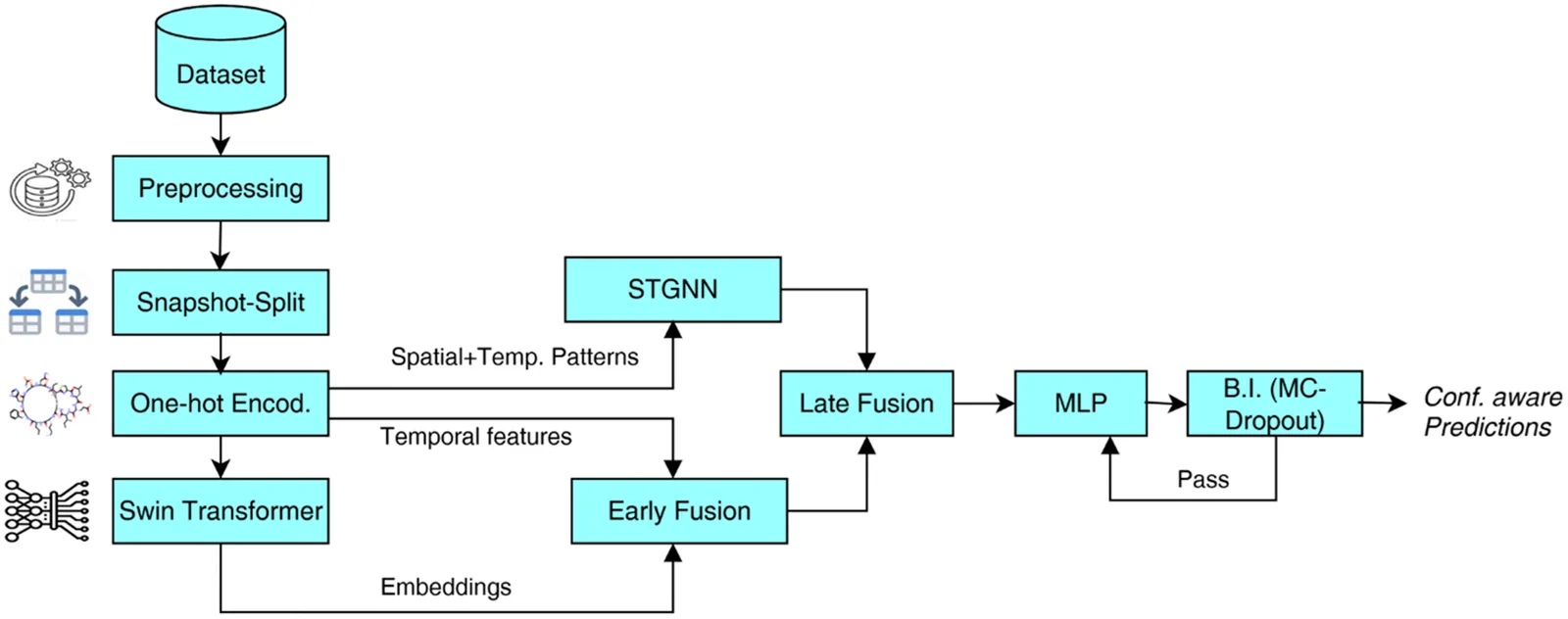
Schematic of the proposed TAG framework. The overall pipeline illustrating the integration of Swin Transformer, STGNN, and Bayesian inference for confidence-aware intrusion detection in CPS.

The architectural design and operational workflow of TAG is presented in Section 4. Sections 5 is about deployment of the model whereas Section 6 describes the implementation details. Evaluation and analysis are presented in Section 7. Lastly, conclusion with future direction is covered in Section 8.

## Literature review

The growing use of CPS across energy, industrial, and IoT-enabled environments introduces several security challenges mainly because of their heterogeneous components, real-time communication, and wide attack surface. Early security solutions relied mainly on rule-based [8] and signature-based IDS approaches [9] to detect intrusions and anomalous behaviours. However, these systems suffer from inherent scalability limitations, since fixed rules cannot easily adapt to evolving or polymorphic attacks. The signature-based detectors fail whenever the attack does not match a previously known pattern.

### Conventional machine learning-based systems

To deal with the challenge associated with rule/signal-based rigid systems, ML methods for intrusion detection have been introduced including spanning supervised, unsupervised, and hybrid methods [4]. Supervised models, trained on labelled datasets, generally achieved higher detection rates than classical IDS because they learn discriminative patterns directly from data. The decision-tree-based methods exhibited low false positive rates on two benchmark datasets, NSL-KDD and CICIDS2017 [10]. In the same vein, Support Vector Machines (SVMs) have been used for a long time as powerful classifiers that build the best separating hyperplanes in high-dimensional feature spaces. SVM was successfully used by Mukkamal et al. [11] in their IDS to get acceptable accuracy on the KDD Cup 99 dataset. Nevertheless, SVM-based systems degrade in the presence of very large-scale or highly imbalanced datasets [11]. The k-Nearest Neighbours (KNN) classifier was also adopted for small- to medium-sized datasets, and the studies [12,13] showed that KNN can enhance detection capability when feature spaces are well engineered. In the studies of [14] and [15] convolutional architectures are used to enhance feature representation and classification performance. The integration of encoding schemes with cluster-based learning is suggested in [16]. Data obfuscation is proposed for protecting sensitive information in [17] where encoding based algorithm is suggested in [18]. With regards to the unsupervised sides, abnormal detection techniques were probed to deal with CPS data that was either unlabelled or weakly labelled. Auskalnis et al. [19] proposed a Local-Outlier Factor (LOF)-based system for detecting abnormal traffic. Fuzzy C-means clustering was used to identify CPS-related attacks [20,21] through a supervised feature-extraction IDS autoencoder. Thakur et al. [22] suggested a generic-specific autoencoder for domain-focused intrusion detection. A high-throughput framework was developed by [23] to extract network outliers in CPS communication. Other studies, including [24,25] used groups of autoencoders and behavioural snapshots to improve detection across different scenarios. Notwithstanding these efforts, ML-based IDS usually work based on the assumption that the data distribution is stationary. For this reason, their performance weakens when new attacks emerge.

### Deep learning-based intrusion detection for CPS

As the complexity of CPS traffic increases, coupled with the growing popularity of IoT/IIoT settings, researchers have started focusing on Deep Learning (DL) methods [3]. This growing attention can be attributed to the reality that these methods are robust and have strong feature-learning ability. To attain better accuracy at a lower computational cost, deep learning has been used with Random Forest [21]. Similarly, Li et al. [26] put forward the DeepFed system, which amalgamated Convolutional Neural Networks (CNNs) with Gated Recurrent Units (GRUs) to detect intrusions in CPS while preserving parameter privacy through the Paillier cryptosystem. The same scholars applied an OPNET- and MATLAB-based solution able to detect heterogeneous attacks with low false alarms.

Regarding intrusion detection specific to IoT, Ge et al. [27] reported accuracies of 99.9% for binary and 99.7% for multi-class classification using a neural network-based framework. A dual-layer detection model is proposed in [28]. The proposed model integrates distributed CNNs with a cloud-hosted LSTM for detecting attacks associated with phishing. Using Bayesian Regularisation with Scaled Conjugate Gradient, the accuracy obtained by [29] was 97%. To further improve detection performance, a number of studies focused on feature-selection strategies. Roopak et al. [30] applied multi-objective optimisation with a non-dominated sorting algorithm, obtaining a ≈99.9% accuracy after reducing the feature dimension. Hong et al. [31] combined Mutual Information (MI), L1, ANOVA, and Chi-square tests to build an effective hybrid feature-selection pipeline for DDoS detection. Thi-Thu-Huong et al. [32] utilised XGBoost to achieve real-time intrusion detection.

### CPS security graph neural networks

For modern CPS attacks, especially those involving interdependent devices and multi-hop communications, Graph Neural Networks (GNNs) have emerged as one of the most suitable approaches [33]. Different from standard ML/DL models operating on feature vectors, GNNs are able to directly model the topological relationships among CPS entities and identify fine patterns of interaction [34]. As a result, numerous advanced GNN-based IDS frameworks have been suggested. Network-flow graphs were built by Pujol-Perich et al. [35] to detect DDoS and activities related to port-scanning. To incorporate time-based changes, KAIROS [36] was suggested as a temporal graph autoencoder. On the other hand, in the GHUNTER system [37] Graph Isomorphism Networks is used to cope with sneaking attacks. Provenance graphs offer a structured representation of system execution and causal dependencies in the intrusion detection research [33]. So, the effective XFedGraph-Hunter system is introduced [38]. This is a federated learning mechanism for GNN-based IDS, which enables collaborative training across distributed CPS nodes. Even though these are useful solutions, they still face challenges associated with communication overhead while also being susceptible to adversarial attacks.

### Unified spatio-temporal design: Limitations and motivation

Despite considerable progress, most available approaches tend to focus on either the space-based structure of the network or the time-based dynamics of events, but not both at the same time. Techniques like KAIROS [36] and GUNTER [37] capture graph structure or temporal outlines independently. This leads to isolated modelling without the ability to completely show the interdependencies in real-world CPS intrusions. Moreover, contemporary GNN/DL-based IDS rarely incorporate uncertainty estimation, even though confidence awareness is essential for decision-making in safety-critical CPS deployments. These are the limitations that encourage the design of an amalgamated framework able to fuse representations from complementary models jointly taking advantage of time-based and space-based features, and augment intrusion prediction with post-hoc Bayesian inference to provide alerts that are confidence-aware. The framework proposed in this work follows exactly this direction, ensuring that complex relational patterns are captured while maintaining reliable classification for CPS security.

To provide a clearer comparative perspective of the related studies, Table 1 summarises key characteristics of existing intrusion detection frameworks discussed in this section. It can be inferred that even though the standard ML and DL models obtain a high level of accuracy in detection, they usually fail when it comes to capturing long-term dependencies or quantifying uncertainty. Recent GNN-based systems improved structural representation but lacked temporal and probabilistic reasoning, motivating the development of the unified TAG framework.

**Table 1:** Summary of representative intrusion detection studies for cyber-physical systems.

Reference	Model	Dataset(s)	Contributions	Limitation
Diwan et al., 2013 [10]	Decision Tree (ML)	NSL-KDD CICIDS2017	Low false- positive rate	Limited generalization
Li et al., 2021 [26]	DeepFed (CNN + GRU)	Custom CPS Dataset	Secure federated intrusion detection	High computational cost
Ge et al., 2021 [27]	Neural Network	IoT Dataset	≈99.9% binary accuracy	Limited interpretability
Pujol-Perich et al., 2022 [35]	GNN (Network Flow Graph)	TON-IoT	DDoS and port-scan detection	Overlooks temporal dynamics
Cheng et al., 2023 [37]	GHUNTER (GIN)	SWaT WADI	Stealth attack modelling	No Bayesian confidence estimation
**Proposed (TAG)**	STGNN + Swin Transformer + Bayesian Inference	UNSW-NB15 TON-IoT SWaT N-BaIoT	Integrated spatio-temporal and confidence- aware intrusion detection	Bridges temporal, spatial and uncertainty modelling gaps

The table summarizes representative intrusion detection approaches for cyber-physical systems, highlighting their underlying models, benchmark datasets, principal contributions, and remaining research limitations.

### Research gap

Although, IDS based on graph neural networks effectively capture structural dependencies but insufficiently model long-range temporal dynamics. Existing DDoS systems based on DL methods mainly focus on cloud or enterprise networks [4] and generally do not model spatial dependencies and temporal evolution simultaneously. On the other hand, transformer-based approaches excel at sequential modelling but provide limited representation of graph topology and inter-entity relationships. Similarly, the contemporary hybrid methods typically rely on simple feature fusion and rarely account for predictive uncertainty. These limitations motivate the proposed TAG framework, which unifies in a single end-to-end architecture the learning of spatio-temporal dynamics, attention-guided feature fusion, and Bayesian uncertainty estimation.

## Preliminaries

Before discussing methodology, the foundational concepts used in this research work are elaborated in this sections.

### Cyber-physical systems

Cyber-Physical System (CPS) is the systematic integration of cyber and physical components [1,14,39]. In a CPS, embedded computers are employed to monitor physical components through networked connections with feedback loops [5]. CPSs are widely used in critical infrastructure domains like autonomous vehicles, smart grids, and industrial control systems. A CPS is formally described as,


$$\begin{array}{c} CPS=(P_{y},\;C_{y},\;N_{c},\;F_{f}) \end{array}$$ (1)

where,

$P_{y}$ represents the physical components, like sensors and actuators,

$C_{y}$ denotes the cyber components, e.g., controllers

$N_{c}$ is the network interconnecting $P_{y}$ and $C_{y}$

$F_{f}$ is the feedback function that serves to couple physical states and cyber decisions

### CPS intrusions

As all CPS exchange information via communication networks, their vulnerability to threats and attacks remain high [5]. Besides adversaries initiated cyber-attacks, the system perimeter may be breached by different vector attacks. Attacks with the intent to gain unauthorized access to the system through its data or device, are called intrusions. Some common intrusions with their possible damage are illustrated in Table 2.

**Table 2:** Intrusion types with their potential targets and associated damage in CPS.

Intrusion	Potential Target	Associated Damage
False Data Injection Attack (FDIA)	Sensor Network	Misleading system state
Sensor Spoofing (Tampering)	Physical interface/ sensors	Incorrect perception
Replay Attack	Network (Passive Capture)	Masking real anomalies
Control Hijacking	PLC/SCADA	Malicious actuator control
Man-in-the-Middle (MitM)	Network switch/router	Command manipulation

The table summarizes common cyber-physical system intrusion types, their primary targets, and the potential operational damage caused by each attack.

### Graph auto-encoder

A Graph Auto-encoder (GAE) class employs encoder and decoder to learn the graph-structure in unsupervised fashion. While preserving the topological attributes, the node features are compressed into a vector of embedding by the encoder. The architecture of graph is then reconstructed by the decoder component in the form of adjacency matrix. This makes the complex task of clustering, and links-prediction more effortless. GAE intends to optimize the reconstruction loss and to encode only those features which are semantically richer.

### Encoder

Encoder is the core component of Graph Attention Networks (GATs) that learns adaptive weights to compute structure aware embeddings for each node in a graph. Typically, the weights are learnt by employing the attention mechanism over the graph topology. Distinct from the conventional architecture of message-passing, encoder attempts to evaluate the importance of each node in the neighborhood (ℵ) prior to aggregation. Next, with the mechanism of neighborhood aggregation illustrated in Fig 2, the encoder maps node $n_{i}\in ℵ$, of features $f_{i}$ to a latent embedding $e_{i}$ of dimension $d;e_{i}\in {\mathbb{R}}^{d}$. The generic form of message passing and aggregation (for the neighborhood of a node $e_{i}:{ℵ}_{i}$) performed by the encoder is illustrated in equation 2,


$$\begin{array}{c} e_{i}^{\left(l+1\right)}=\alpha^{l}\left(e_{i}^{l},\;\beta^{l}\left(\{e_{j}^{l}:j\in {ℵ}_{i}\}\right)\right) \end{array}$$ (2)

**Fig 2:**
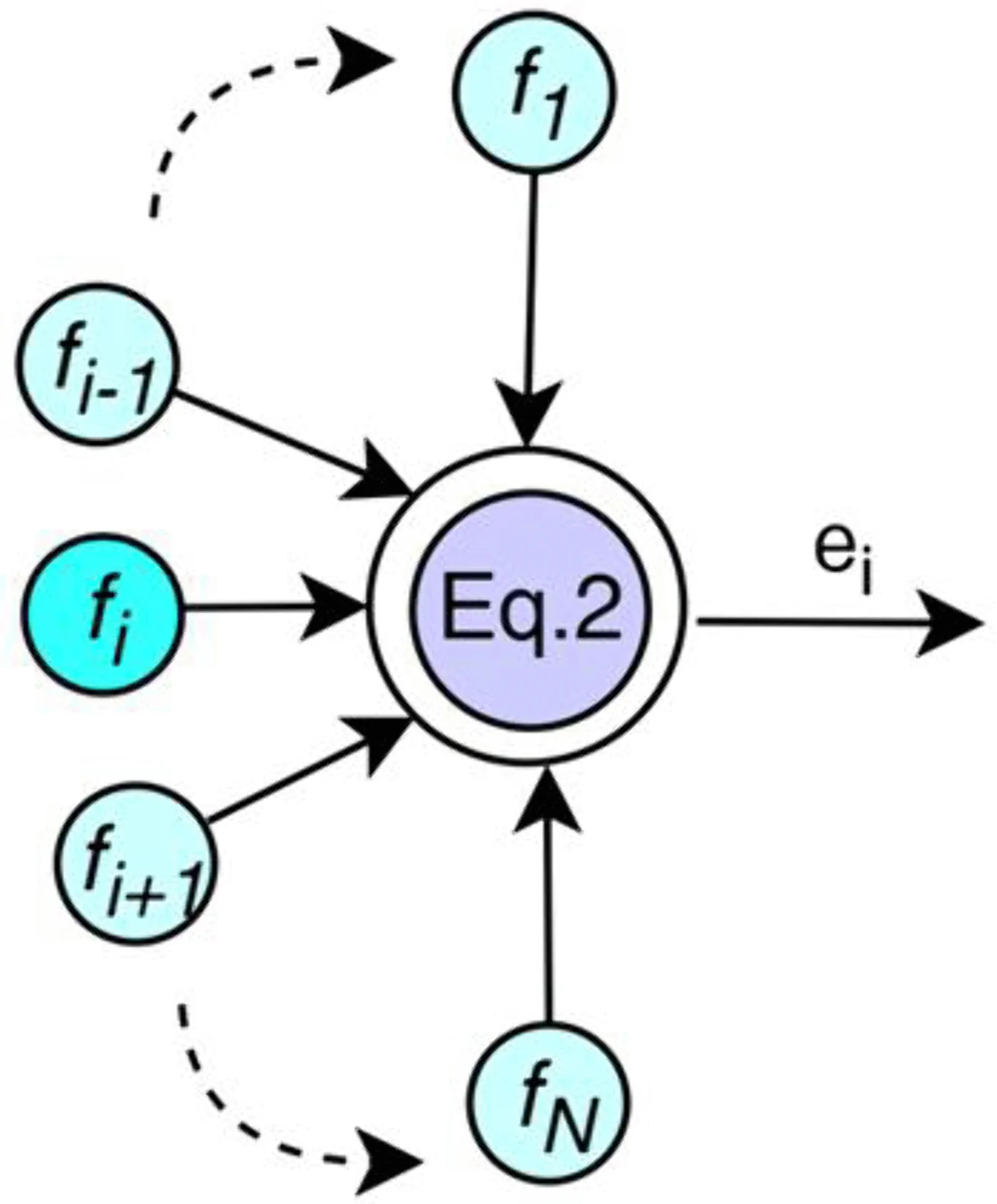
Neighborhood aggregation of feature $f_{i}$.

### Decoder

Role of the decoder; as the name implies, is reconstruction of the node features from the encoder generated latent space. The node embeddings; $\{e_{i}{\}}_{i=1}^{ℵ}$ generated by the encoder for node $n_{i}$ is taken as input to the decoder. With the readout aggregation- *g* followed by MLP- $P_{r}$, a pool vector is mapped to global graph embedding $E_{G}\in {\mathbb{R}}^{d}$ where $G=n_{1},n_{2},.,.,n_{ℵ}$. The decoder then utilizes $E_{G}$ to predict *g*raph-level output- $y^{g}$ as,


$$\begin{array}{c} y^{g}=P_{r}(E_{G}) \end{array}$$ (3)

where,


$$\begin{array}{c} E_{G}=P_{r}\left(R_{\text{out}}\left(\{e_{i}{\}}_{i=1}^{ℵ}\right)\right) \end{array}$$ (4)

However, for link prediction or edge classification, the likelihood of an edge between nodes $n_{i}$ and $n_{j}$ is estimated in a bilinear manner. If *M* denotes the learnable weight matrix, $e_{i}^{T}$ represents the transpose of the embedding of node $n_{i}$, and $e_{j}$ denotes the embedding vector of node $n_{j}$, the prediction is expressed as:


$$\begin{array}{c} y_{\left(i,j\right)}=e_{i}^{T}Me_{j} \end{array}$$ (5)

The working of decoder is illustrated in Fig 3

**Fig 3:**
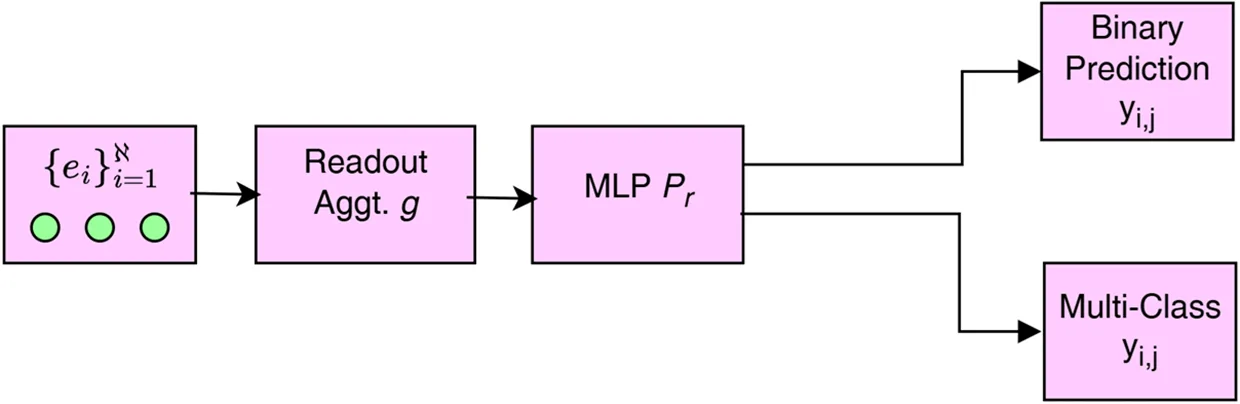
The decoder function for binary and multi-class outputs.

### Graph neural network

Graph Neural Networks (GNN) signify a set of neural architectures that are designed to operate on non-euclidean graph-structured data [40]. A GNN based model takes a graph *G* = (*N*, *E*) with nodes *N* and edges $E:E=\{v_{1},v_{2},..v_{n}\}$. Each node $n_{i}\in N$ is associated with a feature vector $x_{i}\in {\mathbb{R}}^{d}$ which demonstrates attributes of the node. A GNN model aims to learn the latent representation (embedding); $e_{i}^{l}\in \mathbb{R}$ for each node $n_{i}\in N$ at layer *l* by recursively propagating and aggregating information from the local neighborhood ${ℵ}_{i}$. The standard update rule of GNN at layer *l* is given as,


$$\begin{array}{c} e_{i}^{l}=\phi^{l}\left(x_{i}^{\left(l-1\right)},\;\Omega_{j\in {ℵ}_{i}}\;\eta^{l}\left(x_{i}^{\left(l-1\right)},x_{j}^{\left(l-1\right)}\right)\right) \end{array}$$ (6)

where $x_{i}^{\left(l-1\right)}$ represents the previous layer (l−1) feature of node *i*, while ϕ and η are the node updation and message-passing functions respectively. At last layer *L*, a final embedding vector $E_{i}^{L}$ encoding both local structure and semantic context is obtained, see Fig 4. The vector can be further employed for different downstream tasks like, link prediction, node classification, and assigning label to the graph itself. Among the common variants of GNN, the well-known frameworks are Graph Convolutional Network (GCN), Graph Attention Networks (GAT), and Graph Isomorphism Networks (GIN) [40], working of which are illustrated in Fig 5 and their details are presented in S1 Appendix (B).

**Fig 4:**
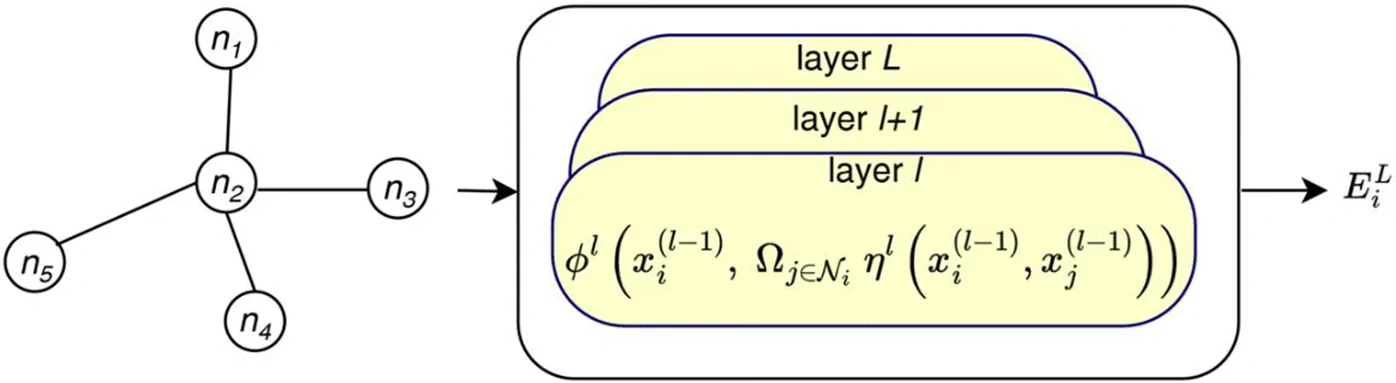
Representation of the function of GNN.

**Fig 5:**
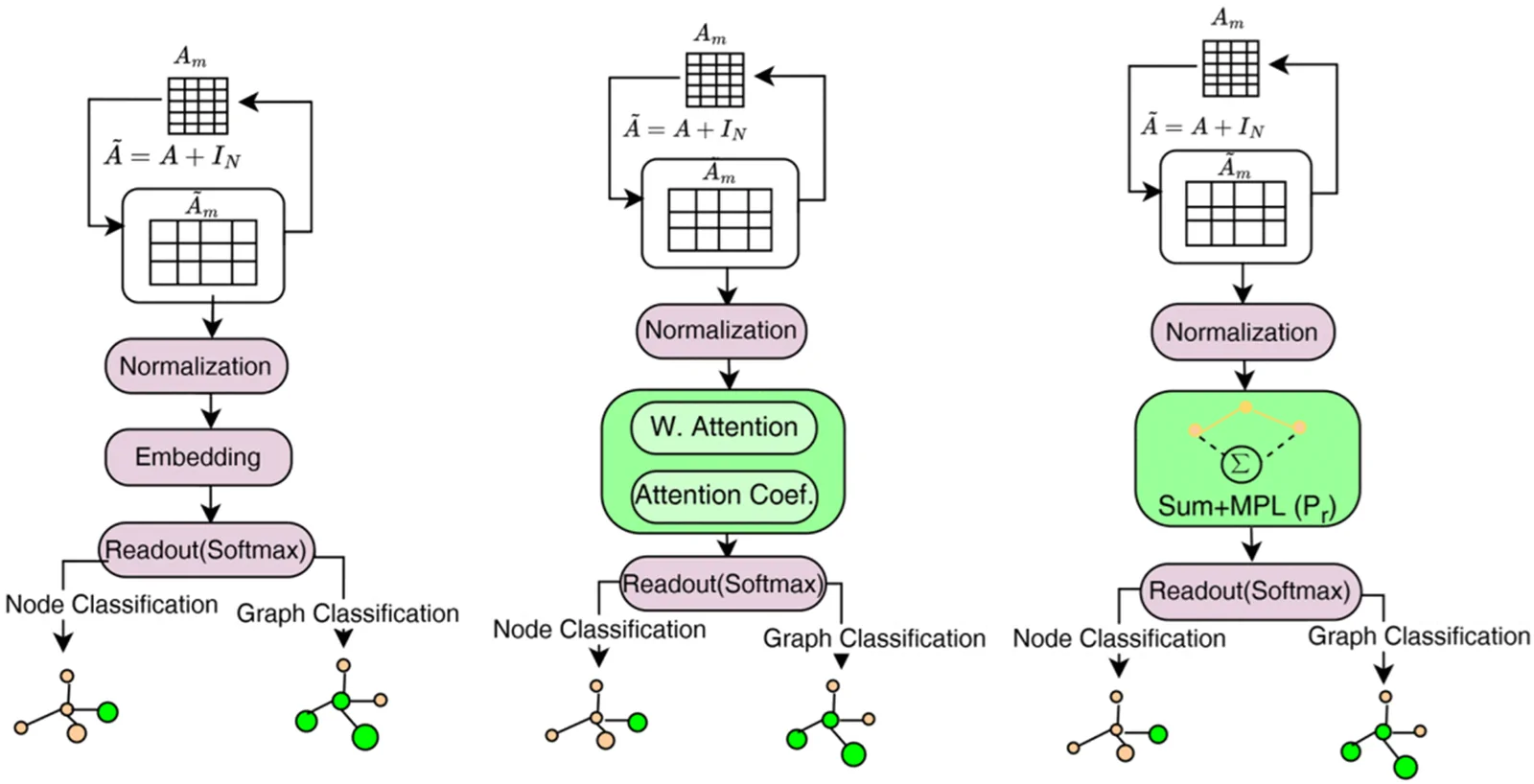
The working mechanism of (a) GCN, (b) GAT, and (c) GIN.

### Swin transformer

The Swin Transformer [39] adopts a hierarchical design that captures both local and global dependencies using a shifted window-based self-attention mechanism [41]. This structure allows efficient learning on high-dimensional data while preserving computational scalability. In addition to applying layer normalization prior to each module, the model performs patch partitioning followed by linear embedding. Let *Z* denote the input feature matrix, where *Z* combines flow-level features ($F_{L}$) and static node-level features ($N_{L}$), expressed as


$$\begin{array}{c} Z=[F_{L}|N_{L}]\in {\mathbb{R}}^{N\times (|F_{L}|+|N_{L}|)}. \end{array}$$ (7)

The input *Z* is divided into non-overlapping patches *p* of size S×S (i.e., p×p). These patches are then embedded into a sequence of feature vectors, producing *Z*_0_ with embedding dimension *d*, where $Z_{0}\in {\mathbb{R}}^{d}$. Further details regarding the flattening process and the self-attention mechanism are provided in S1 Appendix (C).

### Bayesian inference

In safety-critical environments such as CPS, the reliability of model predictions is essential [42]. Bayesian inference offers a principled framework for quantifying uncertainty in neural network models by treating model parameters as probabilistic distributions. Using Bayes’ theorem, the posterior distribution Ω over model parameters π, given observed data *D*, can be expressed as,


$$\begin{array}{c} \Omega (\pi |D)=\frac{\Omega (D|\pi )\;\Omega (\pi )}{\Omega (D)}. \end{array}$$ (8)

The updating of model parameters and intermediate outputs during stochastic forward passes is described in S1 Appendix (D).

## Materials and methods

In the TAG framework, a unified spatio-temporal learning architecture is proposed for intrusion detection in CPS. The temporal representation module based on the Swin Transformer is integrated with STGNN-driven spatial representation module to enable the extraction of temporal and topological information from heterogeneous CPS data. The cutting-edge hierarchical shifted window-based multi-head self-attention is employed to fuse learned learned representation for tracing time-evolving behaviors. The Bayesian uncertainty estimation (MC Dropout) quantifies predictive confidence for reliable risk-aware decision-making. The methodology supports both node-level and graph-level datasets and encompasses data preprocessing, graph construction, feature fusion, and intrusion classification for trustworthy CPS security monitoring.

### Dataset preparation

Like other DL models, data pre-processing is crucial for effective deployment of graph models. Therefore, to have a harmonize format, the datasets are preprocessed prior to their feeding into the training pipeline. Four benchmark datasets, namely SWaT [43], UNSW–NB15 [44], TON–IoT [45], and N-BaIoT [46], are used for training and evaluation. The datasets encompassing a broad spectrum of cyber-attacks and operational behaviours are suitable for assessment of the model under heterogeneous deployment scenarios. To ensure computational feasibility while preserving statistical characteristics, representative subsets are utilized in the development process without altering the underlying class distributions. Following the chronological organization, every 300 consecutive timestamps are aggregated into a graph snapshot. The resulting snapshots are then partitioned into training, validation, and test subsets using a predefined temporal partitioning protocol. The same procedure is adopted for all the datasets and experimentation. Detailed descriptions of the datasets are presented in Tables 3. Missing values are handled, numerical features are normalized, and categorical attributes are encoded prior to sequential snapshots. The process of extracting nodes, edges, and their associated features from the raw datasets is summarized in Algorithm 1.

**Table 3:** Details of the processed datasets and representative attack categories.

Dataset	Features	Normal Samples	Attack Samples	Total Samples	Key Attacks
UNSW-NB15	43	36992	45332	82324	Fuzzers, Analysis, Backdoors, DoS, Exploits, Generic, Reconnaissance, Shellcode, & Worms
TON-IoT	42	50000	161043	211043	Backdoor, DDoS, DoS, Injection, Password, Ransomware, Scanning, XSS, & MitM
SWaT	51	2084	1916	4000	Sensor Spoofing, Actuator Manipulation, Process Disruption, Pump Attacks, Valve Attacks, & Normal Operation
N-BaIoT	42	2000	2000	4000	ACK Flood, SYN Flood, UDP Flood, TCP Flood, Junk Flood, Mirai Scan, Bashlite Scan, & Botnet Activity

The table summarizes the benchmark datasets employed in this study, including the number of features, normal and attack samples, total instances, and representative attack categories used for evaluating intrusion detection performance.

**Algorithm 1: Data Extraction**

**Input:** Flow data file *F*

**Output:** Graph *G* = (*N*, *E*)

1.  Initialize *G* = (*N*, *E*)

2.  **for** each line l∈F **do**

(a) Parse *l* into fields $f_{1},f_{2},...,f_{n}$ based on delimiter

(b) **if** Src∉N **then**

i. N←N∪{Src}

(c) **end if**

(d) **if** Dst∉N **then**

i. N←N∪{Dst}

(e) **end if**

(f) e←(Src,Dst)

(g) E←E∪{e}

3.  **end for**

4.  Return *G* = (*N*, *E*)

### Splitting into snapshots

The flow data in graph-level datasets represents interactions in the form of nodes and edges accompanied by timestamps. Such data are often in log form and needed to be divided into snapshots. Therefore, splitting of the dataset is performed on timestamp basis to have set of events for each specific time range. In the proposed approach, events of 300 timestamps are grouped into one snapshot. For instance, the first snapshot contains events from timestamp 0 to timestamp 299 are covered, see Fig 6. By doing so they are brought into standard format to be applied for graph-level modeling. Algorithm for snapshot generation is illustrated in Algorithm 2.

**Fig 6:**
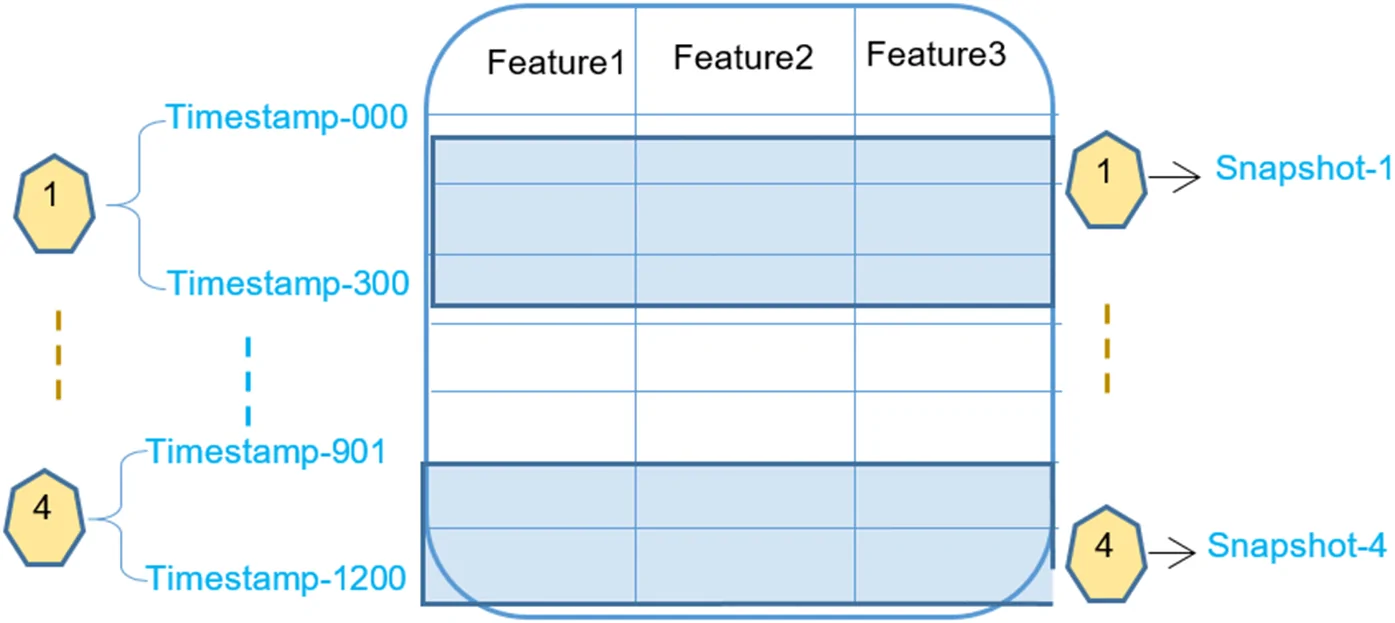
Snapshot splitting of the timestamps.

**Algorithm 2: Splitting Graph Flow-Data into Snapshots**

**Input:** Dataset containing *K* graphs, $K=\{G_{1},G_{2},...,G_{N}\}$; number of snapshots *SH*

**Output:** $SH=\{p_{1},p_{2},...,p_{n}\}$ with node dimension $d_{n}$ and edge dimension $d_{e}$

1.  **for** each input graph $G_{i}\in K$ **do**

(a) Extract nodes $n_{i}\in N$ and edges $e_{i}\in E$

(b) Obtain node and edge features $f_{n}$, $f_{e}$

(c) Extract timestamps $T_{i}=\{t_{e_{1}},t_{e_{2}},...,t_{e_{E}}\}$

(d) $t_{\max}\leftarrow \max(T_{i})$

(e) $d_{e}\leftarrow |f_{e}|$

(f) $d_{n}\leftarrow |f_{n}|$

(g) $\Delta t\leftarrow \frac{t_{\max}}{T}$

2.  **end for**

3.  **for** each snapshot $p_{j}\in SH$ **do**

(a) Initialize empty directed graph $g_{j}=(N_{j},E_{j})$

(b) $t_{\text{start}}\leftarrow (j-1)\Delta t$, $t_{\text{end}}\leftarrow j\Delta t$

(c) $E_{j}\leftarrow \{(n_{\text{source}},n_{\text{target}},t)\in E_{i}|t_{\text{start}}\leq t<t_{\text{end}}\}$

(d) Add $E_{j}$ and corresponding $N_{j}$ to snapshot graph $p_{j}$

4.  **end for**

5.  Return $SH=\{p_{1},...,p_{n}\},d_{n},d_{e}$

### One-hot encoding

All GNNs operate on numerical feature vectors, therefore the flow data need to be in a uniform format. One-hot encoding is a widely use strategy for standardizing the input data. By employing one-hot encoding, the nodes and edges data are molded into a uniform vector structure suitable for the subsequent graph based operations. Each node $n_{i}\in N$, of type $b_{i}^{n}\in C$ is uniquely represented in the resultant vector $O^{n}$, see Fig 7. The process of getting exactly one entry for each node type in the vector of length *d*, is given as,


$$\begin{array}{c} O_{i}^{n}=[\delta (b_{1}^{n},b_{j}^{n}),\;\delta (b_{2}^{n},b_{j}^{n}),\;..,\;\delta (b_{d}^{n},b_{j}^{n}){]}^{C}\in {\mathbb{R}}^{d} \end{array}$$ (9)

**Fig 7:**
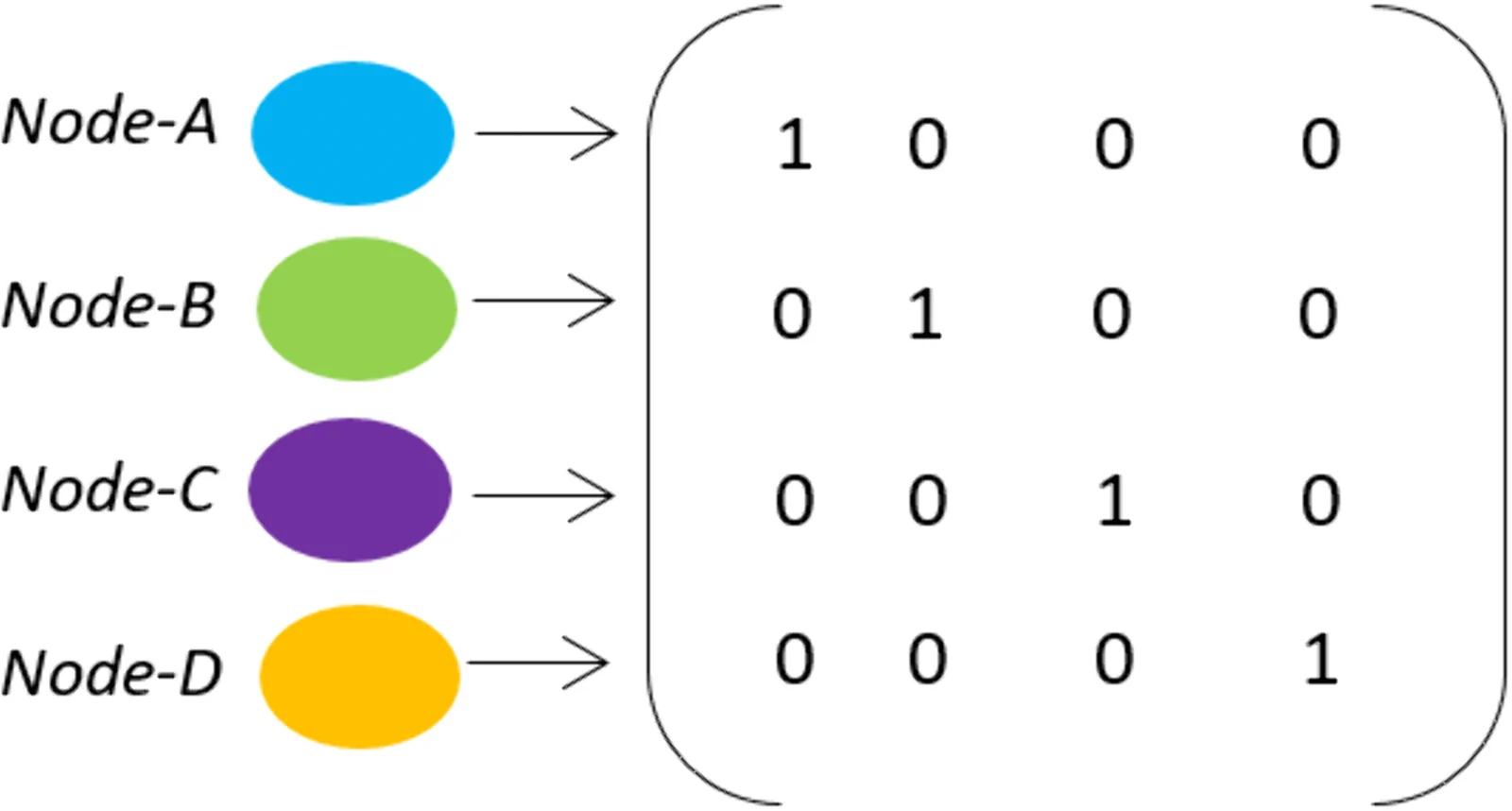
One-hot encoding for the node types.

This ensures unique entry: 1 for each node type and 0 for the rest in the vector. Unique representation of the nodes enables the system to effortlessly distinguish among different CPS nodes like sensor, and activator etc. The same process of one-hot encoding for edge types is given as,


$$\begin{array}{c} O_{i}^{e}=[\delta (b_{1}^{e},b_{j}^{e}),\;\delta (b_{2}^{e},b_{j}^{e}),\;..,\;\delta (b_{e}^{e},b_{j}^{e}){]}^{C}\in {\mathbb{R}}^{e} \end{array}$$ (10)

### STGNN

Spatio-Temporal Graph Neural Networks (STGNNs) [6] is the neural network class to learn and represent graph-structured data. With its effective architecture, STGNN unifies spatial relation with temporal sequence, see Fig 8. The architecture is suitable for CPS where the flow data evolves over time. STGNN is designed to learn spatio-temporal embedding covering the key features including the current and previous neighborhood structure. Thus the embedding can be utilized for the downstream tasks like intrusion detection, node prediction and failure forecasting. If $G_{t}=(N_{t},E_{t})$ is the snapshot of a graph with the features: $X_{t}\in {\mathbb{R}}^{d}:X_{t}=\{x_{1},x_{2},..,x_{n}\}$, STGNN learns the spatio-temporal representation $Q_{t}$ through dedicated spatial and temporal encoders,


$$\begin{array}{c} Q_{t}=f_{T}\left(f_{\chi }(X_{t},G_{t})\right) \end{array}$$ (11)

**Fig 8:**
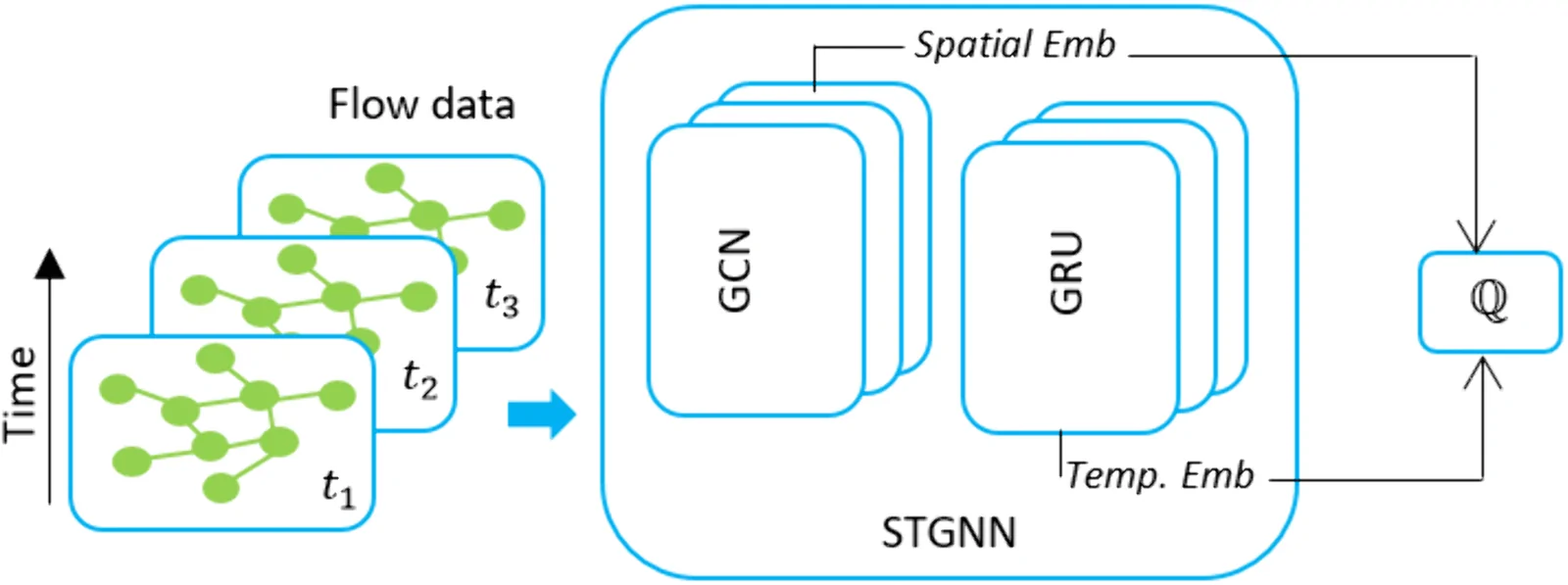
Workflow of the STGNN model.

where $f_{T}$ and $f_{\chi }$ are the temporal and spatial encoding functions respectively.

For spatial modeling, each time the graph structure is extracted by passing the features $x_{i,t}$ of node $n_{i}$ to the graph neural network layer: GCN which employs non-linear activation function. The attention mechanism is implemented through the coefficient $A_{ij}^{t}$, inducing the nodes to dynamically weigh their neighbors at each time step *t*.


$$\begin{array}{c} Q_{i}^{t}=\sum_{j\in {ℵ}_{i}^{t}}A_{ij}\;x_{i}^{t} \end{array}$$ (12)

The $A_{ij}$ is defined as,


$$\begin{array}{c} A_{ij}=\frac{\exp\left(LR\left(v^{t}[Mx_{i}^{t}\;\|\;Mx_{j}^{t}]\right)\right)}{\sum_{j\in {ℵ}_{i}^{t}}\exp\left(LR\left(v^{t}[Mx_{i}^{t}\;\|\;Mx_{j}^{t}]\right)\right)} \end{array}$$ (13)

where *v* is the learnable vector, *M* the weight matrix vector, and ‖ represents the concatenation operation.

For temporal modeling, the Recurrent Neural Network (RNN) is employed with Gated Recurrent Unit (GRU) to extract the node states that evolve with time. The unit takes input feature vector $x_{i}^{t}$ of a node $n_{i}$ at time step *t* and updates its hidden state $e_{i}^{t}\in {\mathbb{R}}^{d}$ by using the vector of the previous hidden s*t*ate $e_{i}^{\left(t-1\right)}$,


$$\begin{array}{c} e_{i}^{t}=GRU(x_{i}^{t},e_{i}^{\left(t-1\right)}) \end{array}$$ (14)

GRU being the variant of RNN, captures the long-term dependencies. Owing to its gating mechanism, GRU is effective in learning the temporal dependencies in dynamic node transitions. Moreover, GRU avoids the common vanishing gradient problem and with its update gate $U_{i}^{t}\in {\mathbb{R}}^{d}$, GRU retains important features from the previous state $e_{i}^{\left(t-1\right)}$ using the sigmoid activation function σ,


$$\begin{array}{c} U_{i}^{t}=\sigma (M_{u}x_{i}^{t}+M_{u-1}e_{i}^{\left(t-1\right)}+b_{u}) \end{array}$$ (15)

where $M_{u}$ and $M_{u-1}$ are the weight matrices of the current and previous state, and $b_{u}$ the learnable bias vector.

Similarly, by its reset gate $R_{i}^{t}$, GRU decides how much of the former information $e_{i}^{\left(t-1\right)}$ be elapsed taking the current input $x_{i}^{t}$ for a node $n_{i}$ at time step *t*,


$$\begin{array}{c} R_{i}^{t}=\sigma (M_{r}x_{i}^{t}+M_{r-1}e_{i}^{\left(t-1\right)}+b_{r}) \end{array}$$ (16)

where $M_{r}$ and $M_{r-1}$ are the learnable weight matrices of the current and previous hidden states.

## Model deployment

The framework proposed contains two core modules: temporal and spatial. In the first module the Swin transformer is exploited to capture the temporal representations from the flow data of a structured network. In the second module, the STGNN models the topological dependencies between flows and hosts of the network. Following preprocessing, the generated feature matrices are transformed into sequential representations and supplied simultaneously to the temporal and spatial modules of the proposed TAG framework. The resulting embeddings are subsequently integrated through the proposed fusion mechanism to produce a unified spatio-temporal representation. Finally, the fused feature space is forwarded to a multilayer perceptron (MLP) classifier for intrusion categorization.

### Temporal module

The Swin transformer encodes each single unidirectional session between two endpoints into an embedding vector. The transformer is designed to apply self-attention across each window $w_{i}$ in a hierarchical way. By taking matrix *X* of time-series features of dimension *d*, of *n* timesteps: $X\in {\mathbb{R}}^{\left(n\times d\right)}$, the shifted-windowing mechanism is employed for cross-window dependencies. In the approach, key role of the transformer is to get the temporally aware embedding. Therefore, the features that may evolve with time like packet count, byte size, and flow duration are taken into consideration. Initially, *X* is transformed to 1D temporal grid by function ε. The result is then forwarded to a positional encoder *p* to have valuable information in sequential order.


$$\begin{array}{c} X^{\prime }=p(\varepsilon (X)) \end{array}$$ (17)

Prior to applying the multi-head self-attention (MSA), $X^{\prime }$ is partitioned into *m* shifted windows $w_{m}$. The windows obtained are non-overlapping with shifting mechanism as illustrated in Fig 9. To model the temporal relationship, window $w_{i}$ is passed to MSA followed by multi-layer perceptron $P_{r}$ for capturing patterns of higher order. Layer normalization $L_{n}$ is also employed to retain standardization in training while achieving temporal embedding *e* of size *d*.


$$\begin{array}{c} e_{i}=MSA(w_{i})+P_{r}\left(L_{n}(w_{i})\right) \end{array}$$ (18)

**Fig 9:**
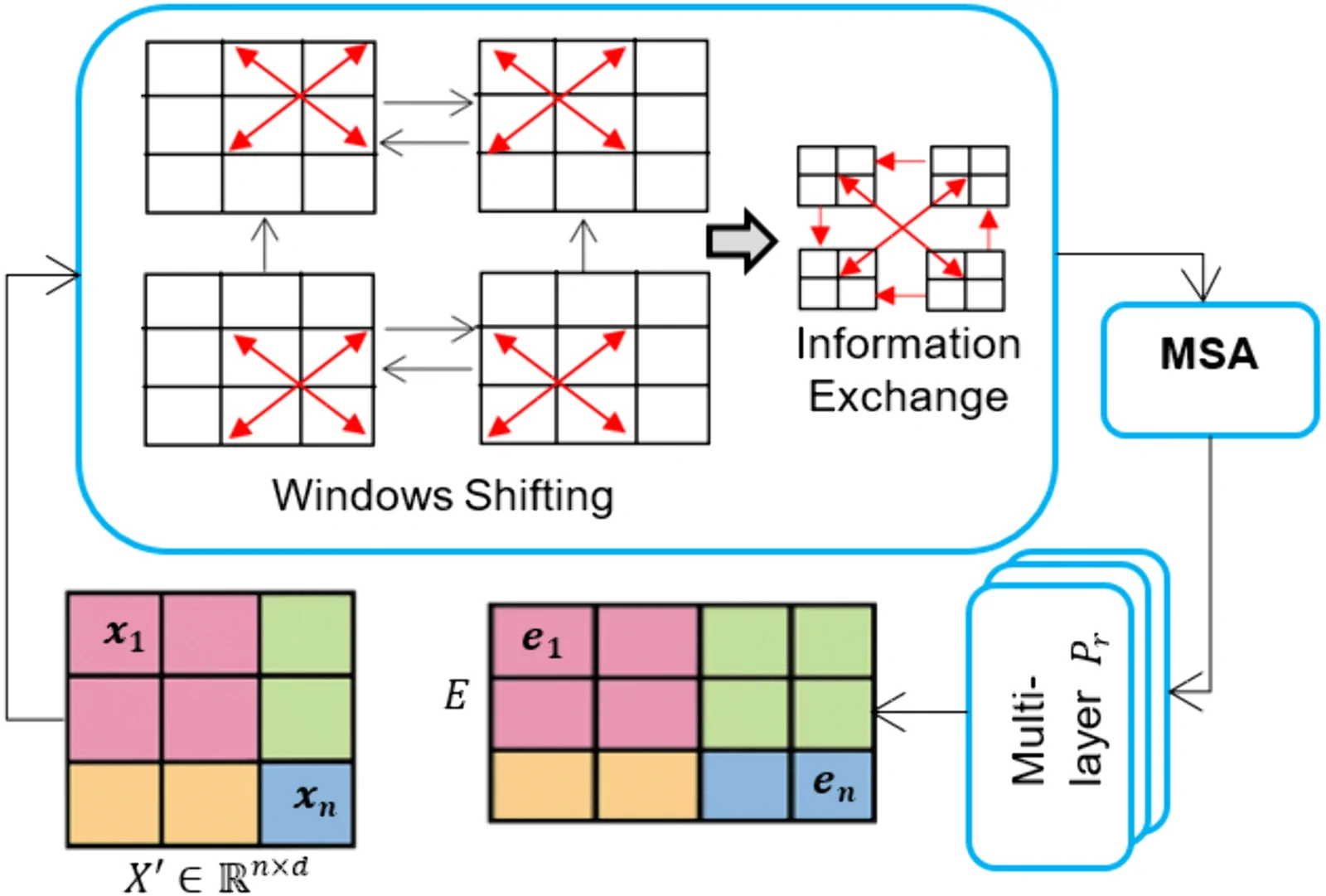
Temporal embedding via shifted-window and multi-head self-attention.

The set of embeddings $E=\{e_{1},e_{2},..,e_{n}\}$ where $e_{i}\in {\mathbb{R}}^{d}$ for each input sequence *i*, is then utilized in different stages of fusion. The vector *E* serves as the node attributes for the dynamic graph which is then fused with the spatial stats of flow data for effective classification.

### Early fusion

Prior to graph based analysis, the static and temporal features are adaptively balanced. Therefore, the flow-level features $f_{i}\in {\mathbb{R}}^{\left(d_{s}\right)}$, with static features size $d_{s}$, are fused with the embedding $e_{i}$ for each node $n_{i}$. Instead of naïve concatenation as practiced in the traditional approaches, attention based fusion is used. The intent behind using attention based fusion is to have more informative features, hence the features $f_{i}$ and embedding $e_{i}$ are projected into a common latent space,


$$\begin{array}{c} f_{i}^{\prime }=M_{f}\cdot f_{i}+b_{f} \end{array}$$ (19)

$$\begin{array}{c} e_{i}^{\prime }=M_{e}\cdot e_{i}+b_{e} \end{array}$$ (20)

where $M_{f}\in {\mathbb{R}}^{\left(d\times d_{s}\right)}$, and $M_{e}\in {\mathbb{R}}^{\left(d\times d_{e}\right)}$, are the trainable weight matrices, where $b_{f}$ and $b_{e}$ being biases respectively. With an attention gate *g*, the most relevant features are captured as,


$$\begin{array}{ccc} & & g_{i}=\sigma \left(A_{a}\cdot \tanh\left(f_{i}^{\prime }+e_{i}^{\prime }\right)+A_{b}\right) \end{array}$$ (21)

where σ is the sigmoid function, while $A_{a}$ and $A_{b}$ are the learnable attention parameters.

By linear interpolation of the gate, fused representation Early Fusion $E_{F_{i}}\in {\mathbb{R}}^{\left(d_{f}\right)}$ of size $d_{f}$ is obtained as,


$$\begin{array}{c} E_{F_{i}}=g_{i}⊙e_{i}^{\prime }+(1-g_{i})⊙f_{i}^{\prime } \end{array}$$ (22)

where ⊙ is the elementwise multiplication.

Thus, the static node level features like packet size, flags, and total packets are jointly captured with temporal embeddings, see Fig 10. The feature vector $E_{F_{i}}$ containing fused contents are utilized in onward graph learning. The process is depicted in Algorithm 3.

**Fig 10:**
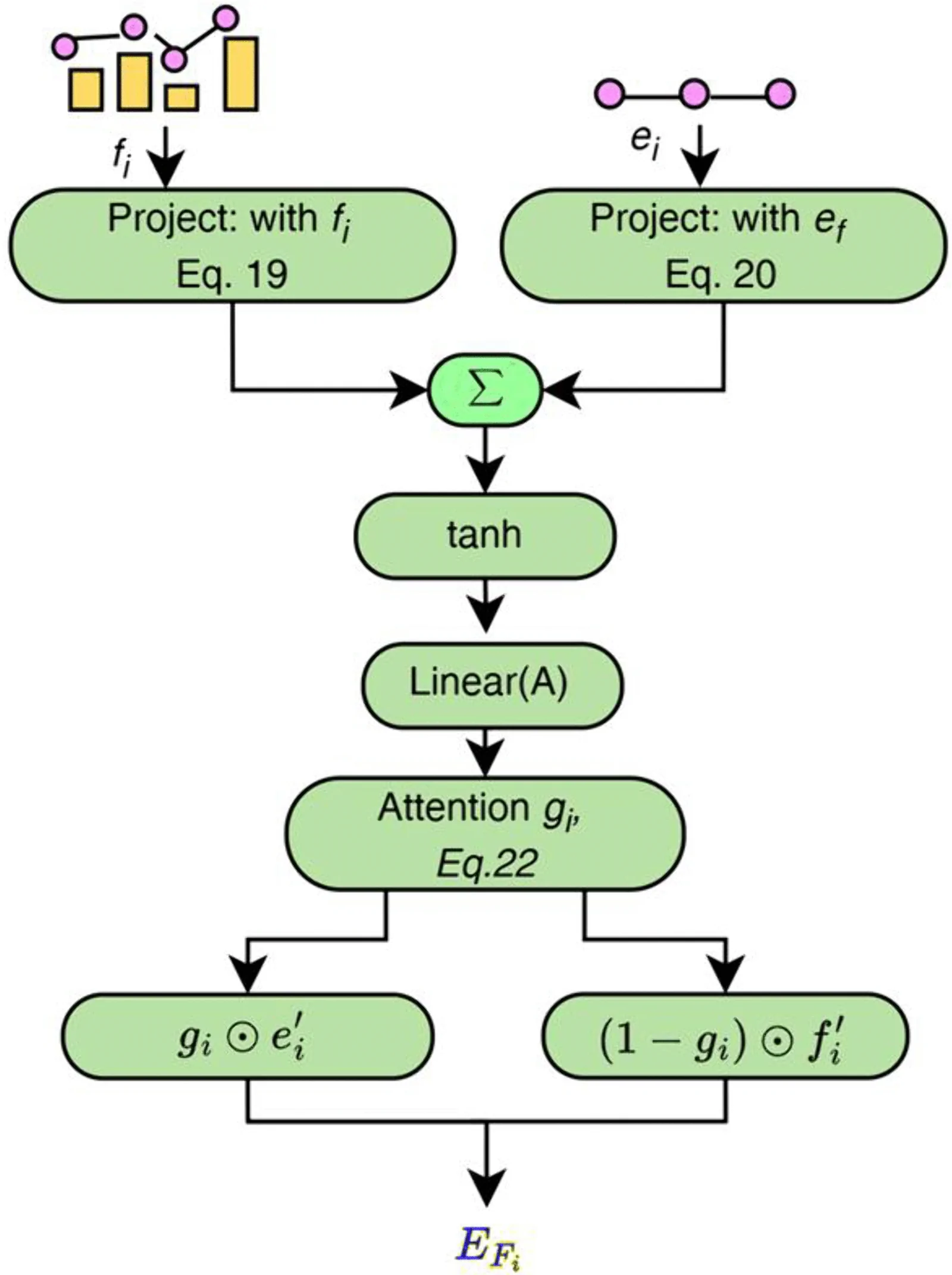
Working of the early fusion module.

**Algorithm 3: Early Fusion**

**Input:** $f_{i}\in {\mathbb{R}}^{d_{s}}$, $e_{i}\in {\mathbb{R}}^{t}$, $M_{f},M_{e}$, $A_{a},A_{b}$

**Output:** $E_{F_{i}}\in {\mathbb{R}}^{d^{\prime }}$

1.  **for** each $n_{i}\in N$ **do**

(a) $f_{i}\leftarrow$ obtain; $e_{i}\leftarrow$ retrieve

(b) $f_{i}^{\prime }=M_{f}\cdot f_{i}$; $e_{i}^{\prime }=M_{e}\cdot e_{i}$

(c) $c_{i}=[f_{i}^{\prime }\|e_{i}^{\prime }]\in {\mathbb{R}}^{2d}$

(d) $g_{i}=\sigma \left(A_{a}\cdot \tanh\left(f_{i}^{\prime }+e_{i}^{\prime }\right)+A_{b}\right)$

(e) $E_{F_{i}}=g_{i}⊙e_{i}^{\prime }+(1-g_{i})⊙f_{i}^{\prime }$

(f) **return** $E_{F_{i}}$

2.  **end for**

### Spatial module

A graph *G* representing the structure of CPS is defined by a set of nodes *N* and edges *E*, such that *G* = (*N*, *E*). The network entities, such as sensors and hosts, are represented by *N*, while *E* denotes the interconnections among these entities. Each node in *N* is enriched with flow statistics, represented in the form of feature vectors. Likewise, edges in *E* capture contextual dependencies between connected nodes, which may correspond to adjacency, direct communication, or statistical similarity.

STGNN is specifically designed to learn relationships among network entities. The model is typically employed to capture spatial dependencies along with temporal dynamics. Given its suitability for learning context-aware representations of complex CPS topologies [46], it is incorporated into the TAG framework. Using STGNN, a high-level embedding $e_{i}^{L}$ is learned for each node $n_{i}\in N$, where each node is initially associated with a feature vector $X_{i}\in {\mathbb{R}}^{d}$.

Through message passing at each layer *l*, the representation of node $n_{i}$ is updated by aggregating feature information from its neighboring nodes. Let ${𝒩}_{i}=\{n_{1},n_{2},...,n_{k}\}$ denote the neighborhood of $n_{i}$. The general form of message passing, using intermediate embeddings $e_{i}^{l}$ at layer *l*, is expressed as:


$$\begin{array}{c} e_{i}^{\left(l+1\right)}=\gamma \left(M^{l}\cdot \alpha \left(\{e_{j}^{l}:j\in {𝒩}_{i}\}\cup \{e_{i}^{l}\}\right)\right) \end{array}$$ (23)

where $e_{i}^{l}$ denotes the node representation at layer *l*, $M^{l}$ is the weight matrix, ${𝒩}_{i}$ represents the set of neighboring nodes, γ is a nonlinear activation function, and α is a permutation-invariant aggregation function. This property a*l*lows STGNN to jointly capture spatial interactions and time-evolving patterns.

To comprehensively model spatial dependencies among CPS entities, a directed communication graph is constructed with an adjacency matrix $A_{m}\in {\mathbb{R}}^{N\times N}$, such that:


$$\begin{array}{c} G=(N,E,A_{m}) \end{array}$$ (24)

At each time step *t*, spatial features $S_{i,t}\in {\mathbb{R}}^{d}$ derived from the flow stream are provided as input to the STGNN. The model propagates and aggregates these features across neighboring nodes. Structural dependencies are learned through convolutional operations. At layer *l*, the feature representation of node $n_{i}$ is updated by combining information from its neighborhood ${𝒩}_{i}$. The resulting spatia*l* embedding $\chi_{i}$ is given by:


$$\begin{array}{c} \chi_{i}=\sigma \left(\sum_{j\in 𝒩(i)}A_{m_{ij}}\;w^{l}\;S_{j}^{\left(l-1\right)}+b^{l}\right) \end{array}$$ (25)

where σ is a non-linear activation function, and $w^{l}$ and $b^{l}$ represent the weight matrix and bias at layer *l*, respectively.

For temporal modeling, each node $n_{i}\in N$ is initialized with a feature representation $T_{i}\in {\mathbb{R}}^{d}$ derived from early fused embeddings $E_{F_{i}}$. The embedding is iteratively updated and aggregated at each layer:


$$\begin{array}{c} T_{i}^{l}=STGNN(e_{i}^{\left(l-1\right)},A_{m},E_{F_{i}}) \end{array}$$ (26)

With temporal attention, the hidden representations of nodes $n_{i}$ and $n_{j}$, having degrees $d_{i}$ and $d_{j}$, respectively, are updated at layer *l* as follows:


$$\begin{array}{c} e^{\left(l+1\right)}=\sigma \left(\sum_{j\in 𝒩(i)}\frac{1}{\sqrt{d_{i}d_{j}}}\;A_{m_{ij}}\;e_{j}^{l}\;w^{l}+e_{i}^{l}\right) \end{array}$$ (27)

where $A_{m_{ij}}$ denotes the adjacency relationship, σ is the activation function, and *e* represents the embedding vector.

### Late fusion

To achieve adaptive fusion, an attention-based mechanism is employed to combine the outputs of the Swin Transformer and STGNN. For each node $n_{i}$, where i={1,2,3,...,N}, the embedding $E_{F_{i}}$ obtained from the transformer is fused with the high-level spatio-temporal embedding $Q_{i}$. Since certain intrusions exhibit either temporal or structural distinctiveness, simple concatenation of $E_{F_{i}}$ and $Q_{i}$ may not be sufficient. Therefore, an attention mechanism is adopted to dynamically assign importance to each modality.

Through this mechanism, different weights are assigned to features based on their relevance. A query-key-value attention formulation is utilized, enabling the model to focus on the most informative modality for each flow instance. Prior to fusion, the temporal embedding from the Swin Transformer, $E_{F_{i}}$, and the spatial embedding from STGNN, $\chi_{i}$, are aligned within a shared attention space 𝒟 using linear projections.

The query (*q*), key (*k*), and value (*v*) vectors are computed as follows:


$$\begin{array}{c} q_{T}^{i}=M_{q}Q_{T}^{i},\;k_{S}^{i}=M_{k}Q_{S}^{i},\;v_{S}^{i}=M_{v}Q_{S}^{i} \end{array}$$

$$\begin{array}{c} q_{S}^{i}=M_{q}Q_{S}^{i},\;k_{T}^{i}=M_{k}Q_{T}^{i},\;v_{T}^{i}=M_{v}Q_{T}^{i} \end{array}$$

where $M_{q}$, $M_{k}$, and $M_{v}$ are learnable projection matrices, and $Q_{S}^{i}$ and $Q_{T}^{i}$ denote spatial and temporal feature vectors, respectively.

The attention coefficient $A_{i}\in [0,1]$ for each node $n_{i}\in N$ is computed using scaled dot-product attention:


$$\begin{array}{c} A_{i}=Softmax\left(\frac{\langle q_{T}^{i},k_{T}^{i}\rangle }{\sqrt{s_{A}}}\right) \end{array}$$ (28)

where $s_{A}$ is a scaling factor used for stabilizing gradients.

This coefficient reflects the relative importance of temporal versus spatial information, allowing the model to emphasize the most relevant modality. Through cross-attention, temporal features are modulated with spatial representations to jointly capture dependencies in network traffic sequences.

The final fused embedding vector $\xi_{T}^{i}\in {\mathbb{R}}^{d}$ for node $n_{i}$ is computed as:


$$\begin{array}{c} \xi_{T}^{i}=A_{i}\cdot v_{T}^{i}+(1-A_{i})\cdot E_{F_{i}} \end{array}$$ (29)

where $v_{T}^{i}$ represents the value vector from STGNN, and $E_{F_{i}}$ corresponds to the embedding from the Swin Transformer. As illustrated in Fig 11, the fused temporal vector $\xi_{T}^{i}$ and spatial vector $\chi_{i}$ are concatenated using bilinear pooling to obtain a joint representation $H_{i}$:


$$\begin{array}{c} {ℍ}_{i}=[\xi_{T}^{i}\;||\;\chi_{i}]\in {\mathbb{R}}^{d_{T}+d_{Q}} \end{array}$$ (30)

**Fig 11:**
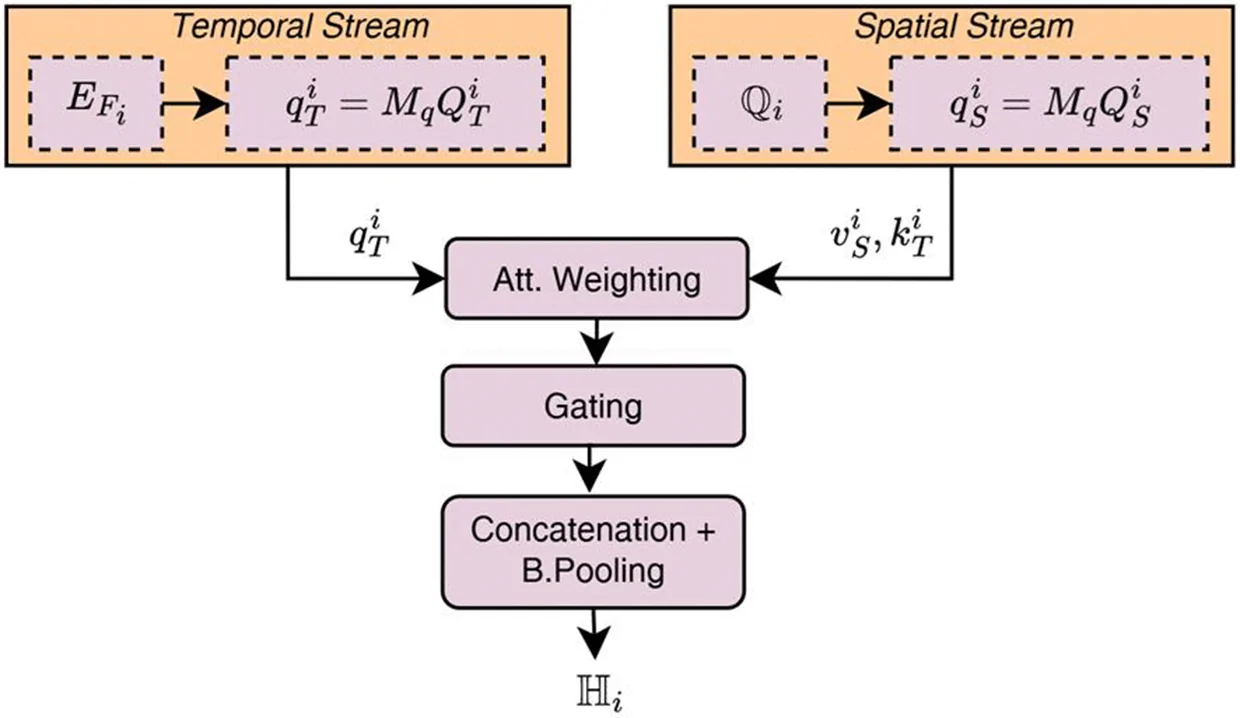
Schematic of the late fusion mechanism.

### Intrusion detection

Labeled training samples are utilized to learn discriminative representations of normal and malicious behavior. Instead of relying on handcrafted features, TAG jointly learns temporal dependencies through the Swin Transformer and spatial relationships through the STGNN. By taking the fused temporal embeddings and spatial patterns, the model performs effortless classification. By exploiting the fused learned embeddings, not only intrusions but any outlier deviating from normal behavior are easily detect. Hence, the joint feature representation $H_{i}$ is passed through a fully connected feed-forward network to produce logits $y_{i}\in {\mathbb{R}}^{C}$, where *C* denotes the number of classes.


$$\begin{array}{c} y_{i}=\omega_{2}\cdot \sigma (\omega_{1}\cdot H_{i}+b_{1})+b_{2} \end{array}$$ (31)

where $\omega_{1}$ and $\omega_{2}$ are the weight matrices of the first and final layers, respectively, *b*_1_ and *b*_2_ are the corresponding bias terms, and σ denotes the activation function. For graph-level classification, $y_{i}\in \{0,1\}$, where 0 represents benign traffic and 1 indicates intrusion. In contrast, for node-level inputs, the model predicts a class label from multiple categories.

### Post-hoc inference

To assess the reliability of intrusion detection, Bayesian inference is employed. Instead of relying solely on deterministic probabilistic outputs, an MC Dropout layer is applied after the MLP. Uncertainty is estimated by passing the final logits to a Bayesian inference layer. This post-hoc use of Bayesian inference avoids modifying the core architecture of the Transformer and STGNN, thereby maintaining modularity and computational efficiency. As a result, the framework produces predictions along with uncertainty estimates, which is particularly important for safety-critical applications. The output of the MLP, $y_{i}\in \{0,1\}$, is provided as input to the MC Dropout-based Bayesian approximation $B_{\theta }$. For each stochastic forward pass $\vartheta_{t}$, a prediction $y^{\left(\vartheta_{t}\right)}$ is obtained. Over *T* such passes, a set of predictions is generated:


$$\begin{array}{c} \{y^{\left(\vartheta_{1}\right)},y^{\left(\vartheta_{2}\right)},...,y^{\left(\vartheta_{T}\right)}\}. \end{array}$$ (32)

Each prediction $y^{\left(\vartheta_{t}\right)}$ is computed using a different dropout mask, resulting in a distribution over outputs. The predictive mean is then calculated as:


$$\begin{array}{c} \mu_{y}=\frac{1}{T}\sum_{t=1}^{T}y^{\left(\vartheta_{t}\right)}. \end{array}$$ (33)

Using $\mu_{y}$ as the final prediction, the epistemic uncertainty is quantified via the predictive variance:


$$\begin{array}{c} \sigma_{y}^{2}=\frac{1}{T}\sum_{t=1}^{T}{\left(y^{\left(\vartheta_{t}\right)}-\mu_{y}\right)}^{2}. \end{array}$$ (34)

The variance $\sigma_{y}^{2}$ reflects the model’s confidence in its prediction: lower values indicate higher confidence, while higher values correspond to greater uncertainty. The final output is expressed as a tuple $\left(\mu_{y},\sigma_{y}^{2}\right)$, enabling confidence-aware decision-making and facilitating real-time threat response modeling.

## Implementation and reproducibility details

The TAG framework integrates Swin Transformer and Spatio-Temporal Graph Neural Network (STGNN) for relational and temporal modeling. An end-to-end joint training strategy is followed for effective detection of intrusions whereas Bayesian inference module is employed for uncertainty estimation. The framework is implemented in Python with the support of various open source libraries for graph data manipulation and neural network operations. A virtual environment was created for training and testing of the model with NVIDIA GPU acceleration. Machine equipped with Intel Core i5-12400F CPU, 16GB RAM and 12 GB VRAM NVIDIA GPU was used to implement and evaluate the system. Flow data from the four datasets containing nodes and edges based communication attributes is used for implementation and evaluation of the framework. All the datasets are partitioned into training, validation, and testing subsets using the customary 70:20:10 ratio. The experimental configuration enables the proposed framework to effectively learn the structural dependencies present in the datasets along-with the node type, see Fig. 12. The issue of class imbalance is properly managed by class-weighted cross-entropy approach where the influence of skewed class distributions is mitigated during model optimization. Comparatively greater penalty is assigned to classification errors associated with under-represented intrusion categories To facilitate reproducibility, details about the implementation settings are summarized in Table 4 Identical hyperparameters were adopted for all the datasets whereas validation loss based early stopping was used to prevent overfitting. “‘latex”’

**Fig 12:**
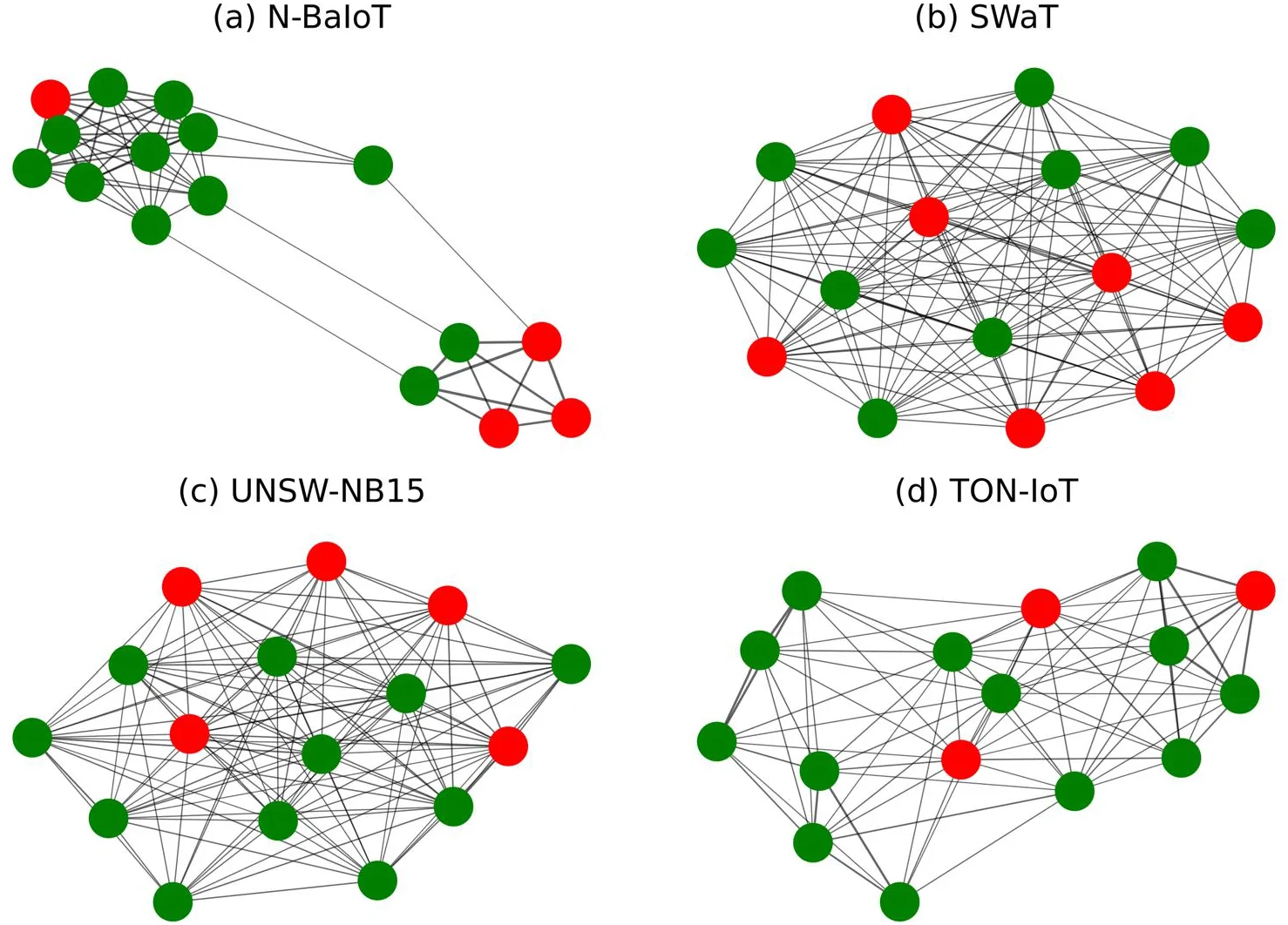
The learned spatial graphs for a) N-bIoT, b) SWaT, c) UNSW-NB15, and d) TON-IoT. Green nodes represent benign entities, whereas red nodes denote attack-related entities.

**Table 4:** Implementation and reproducibility details of the framework.

Parameter	Value
Optimizer	AdamW
Learning Rate	$1\times 10^{-4}$
Batch Size	16–64
Number of Epochs	50
Weight Decay	$1\times 10^{-5}$
Dropout Rate	0.30
Hidden Dimension	128
Attention Heads	4
Swin Window Size	4
MC-Dropout Forward Passes	30
Early Stopping Patience	10 epochs
Loss Function	Cross-Entropy Loss
Random Seed	42
GPU	NVIDIA Tesla T4
CUDA Version	12.1

### Training strategy

To have adaptive training, the approach of supervised learning with mini-batch optimization is followed where in each epoch, training samples were shuffled to reduce ordering bias. Performance of TAG is influenced by optimization hyperparameters selected through empirical validation to ensure stable convergence, and robust generalization across the heterogeneous CPS datasets. Rectified Linear Unit (ReLU) activations are utilized within intermediate layers for the extraction of nonlinear feature, whereas the Softmax function is used at the output layer to generate class probabilities. With the use of Adam optimizer, optimization of adaptive learning-rate and stable convergence is ensured. Initially learning rate is set to ($1\times 10^{-4}$), while a weight decay factor of ($1\times 10^{-5}$) to mitigate overfitting and improve generalization. Network parameters are updated through backpropagation by minimizing the cross-entropy loss function. The final hyperparameter configuration was selected based on validation performance and computational efficiency, inference speed, and convergence stability. Keeping the core architectural components and global settings unchanged, size of mini-batch is adjusted according to the scale of each dataset, as presented in Table 5. The intent behind was to achieve stable optimization and cost-effective resource utilization.

**Table 5:** Dataset-specific training hyperparameters of TAG.

Dataset	Batch Size	Epochs	Early Stopping	Learning Rate	Hidden Dimension	Classes
TON-IoT	64	50	Patience = 10	$1\times 10^{-4}$	128	10
UNSW-NB15	32	50	Patience = 10	$1\times 10^{-4}$	128	10
SWaT	16	50	Patience = 10	$1\times 10^{-4}$	128	2
N-BaIoT	16	50	Patience = 10	$1\times 10^{-4}$	128	5${\;}^{*}$

## Evaluation and analysis

In order to systematically assess performance of the TAG, evaluation by graph-level datasets and node-level datasets was conducted separately. The evaluation is conducted by taking test sets from each of the four different datasets. The sets contain data points unknown to the TAG system. After spatio-temporal feature extraction and fusion, final classification decision is generated by the multilayer perceptron (MLP) classifier. For binary classification, the output layer produces class probabilities through the Softmax activation function where a class with the highest posterior probability is predicted:


$$\begin{array}{c} C_{l}=\text{arg}\max_{i}y_{i} \end{array}$$ (35)

Testing of the system by clustered data of various snapshot is performed. As a result, a node or subgraph is either predicted benign or otherwise marked as intrusion. Performance of the system is quantified using the standard metrics of Accuracy, Precision, Recall, F1-score, True Positive Rate (TPR), False Positive Rate (FPR), and ROC-AUC. Reduceing the possibility of training variability, all experiments are repeated multiple times and results of each metric are presented in terms of mean (μ) and standard deviation (σ). As shown in Table 6, promising precision, recall and F1 scores are obtained for both node-level and graph-level intrusion detection.

**Table 6:** Outcomes of the evaluation.

Representation Level${\;}^{*}$	Dataset	Accuracy (μ±σ)	Precision (μ±σ)	Recall (μ±σ)	F1-Score (μ±σ)	FPR (μ±σ)
Graph Level	TON-IoT	0.990±0.008	0.990±0.016	0.990±0.007	0.990±0.017	0.010±0.001
	UNSW-NB15	0.970±0.076	0.960±0.009	0.940±0.031	0.970±0.008	0.040±0.005
Node Level	SWaT	0.980±0.008	0.980±0.007	0.970±0.007	0.980±0.008	0.030±0.004
	N-BaIoT	0.970±0.009	0.970±0.015	0.950±0.012	0.980±0.007	0.050±0.005

*Graph-level and node-level refer to the structural granularity of the input representation used for learning rather than to distinct categories of cyber intrusions.

Among the graph-level datasets, TAG achieves noteworthy performance on TON-IoT. A slightly lower F1 score observed for UNSW-NB15 which is due to the large number of node types, features and edges in the dataset. For node-level intrusion detection strong efficacy is attained on SWaT, see Fig. 13 for the obtained FN and TP scores of the SWaT and ToN-IoT. The Receiver Operating Characteristic (ROC) curves obtained for all the datasets is shown in Fig. 14 whereas the aggregated Area Under the Curve (AUC) indicating promising classification capabilityis of the model is illustrated in Fig. 15.

**Fig 13:**
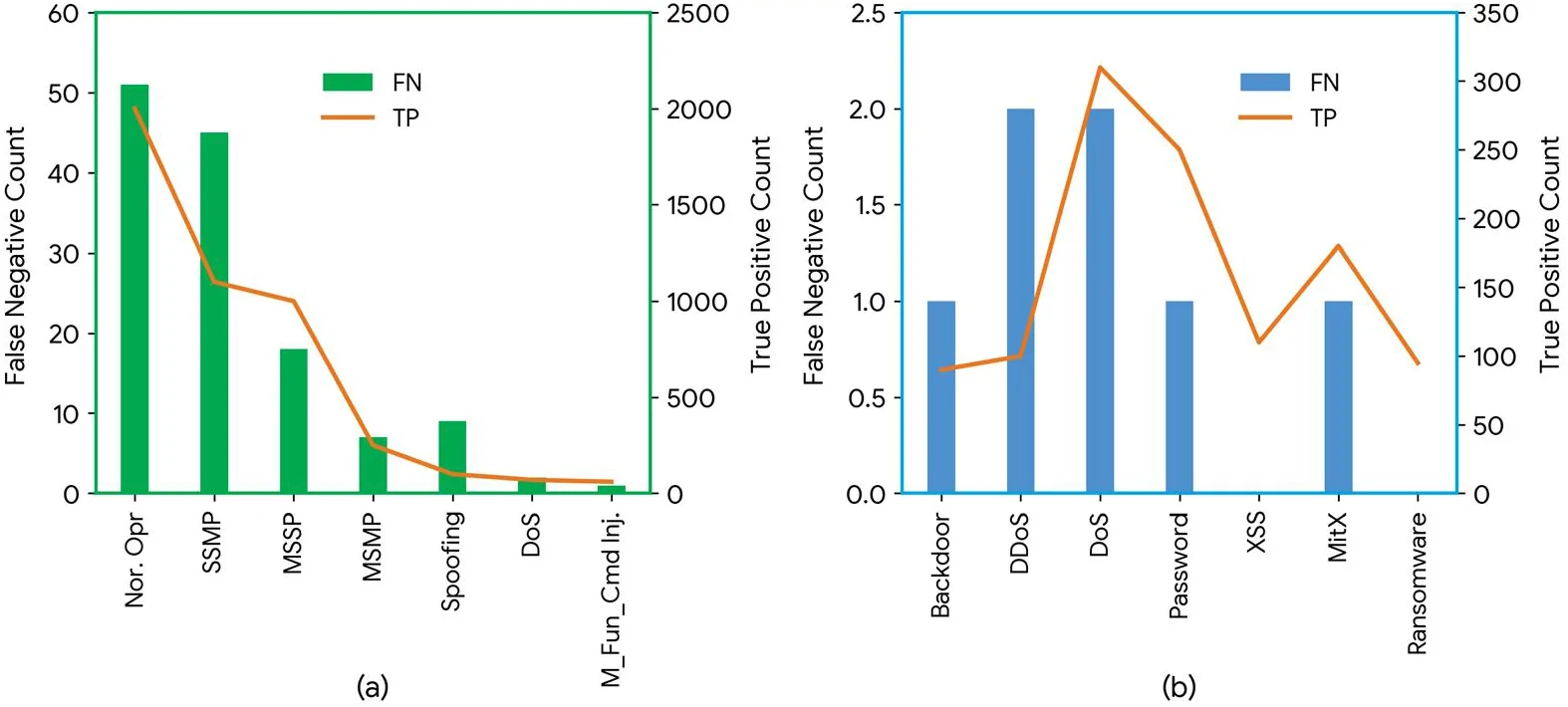
The FN and TP scores obtained for the (a) SWaT, and (c) TON-IoT datasets.

**Fig 14:**
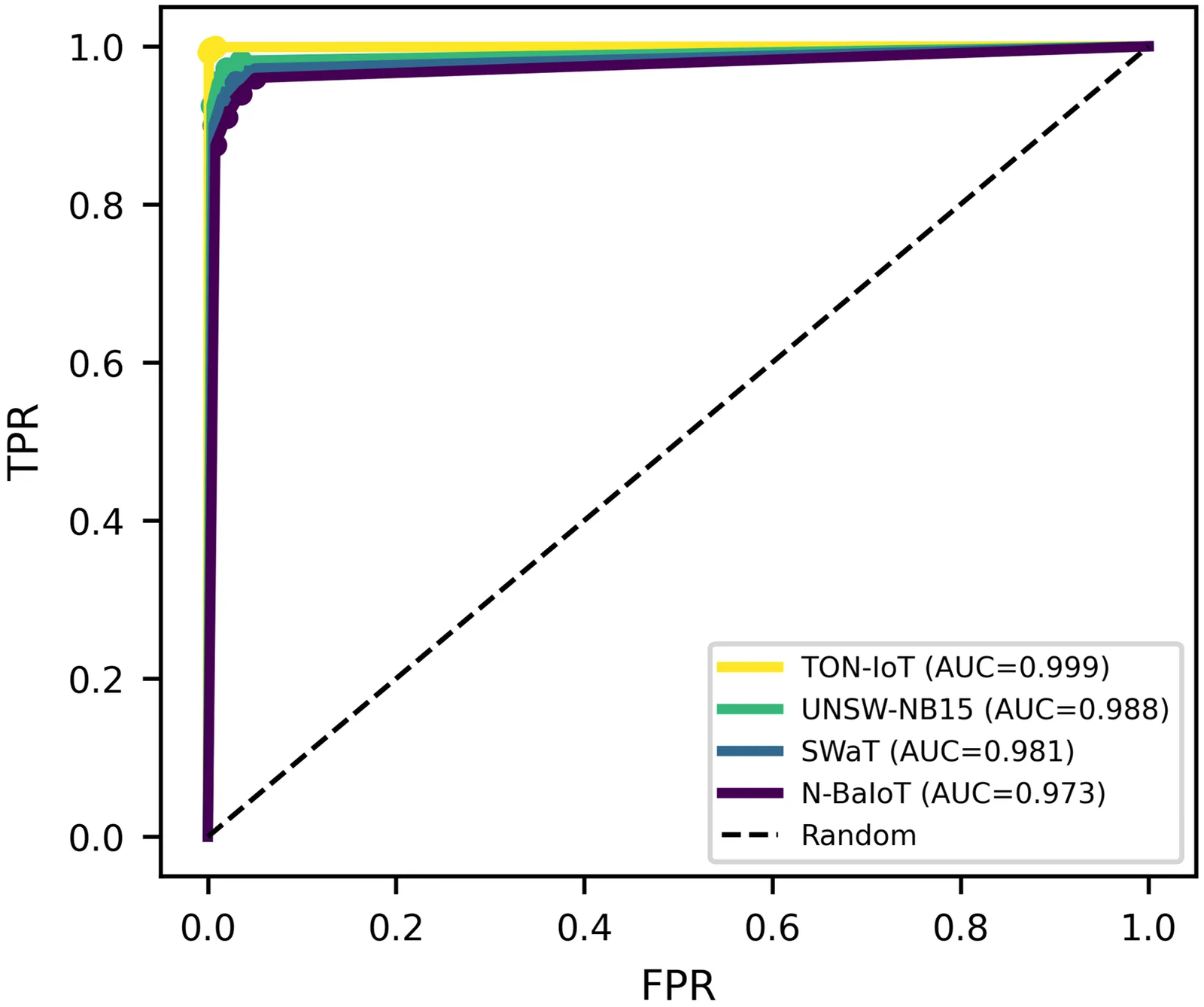
ROC curves, with AUC values across the benchmark datasets.

**Fig 15:**
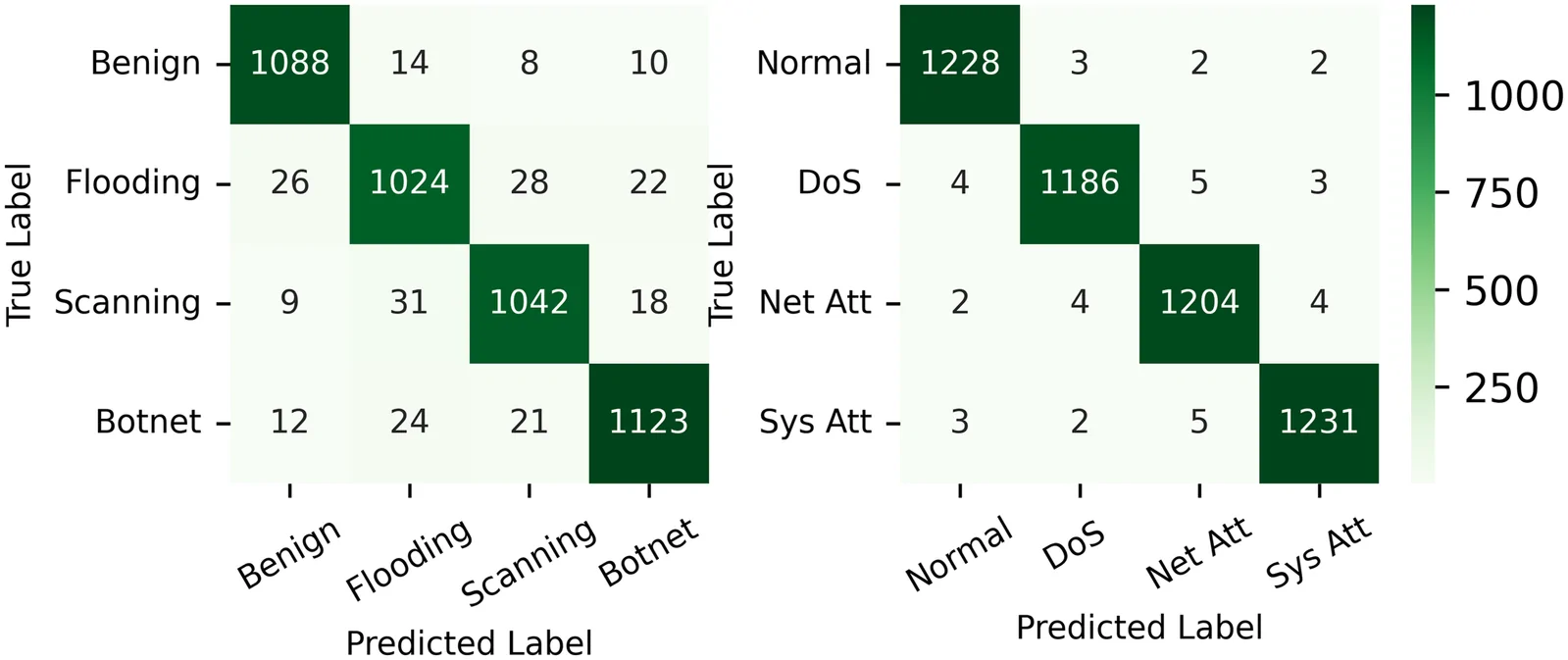
The obtained confusion matrices for (a) Ba-IoT and (b) TON-IoT datasets with number of instances by the side color-bars.

Considering the multi-class classification, a lower F1 score with normal TPR is obtained for the node-level dataset, see Fig 15(a). The comparatively lower performance is attributed to the presence of subtle attack patterns closely resembling the normal variations. However, a notable F1 score of 0.99 is obtained for the TON-IoT dataset, see Fig 15(b). Despite the marked variations, TAG maintained high predictive performance over the evaluated datasets, particularly, the F1-scores and ROC-AUC values which ranged between 0.97 & 0.99. Moreover, the low standard deviations over repeated experimental runs indicate that the hybrid architecture exhibits stable optimization behavior. The absence of significant performance drop across both the CPS datasets further demonstrates generalization capability of the spatio-temporal learning proposed.

The binary confusion matrices presented in Fig 16 further validate robustness of the framework. For investigating discriminative capability of TAG, the feature embeddings produced by the proposed TAG framework is visualized using the t-distributed Stochastic Neighbor Embedding (t-SNE). The resulting projections, see Fig 17, reflect strong inter-class separation capability of the system. The observed clear separation. Overall results confirm the robustness and generalization capability of the proposed framework across heterogeneous CPS environments.

**Fig 16:**
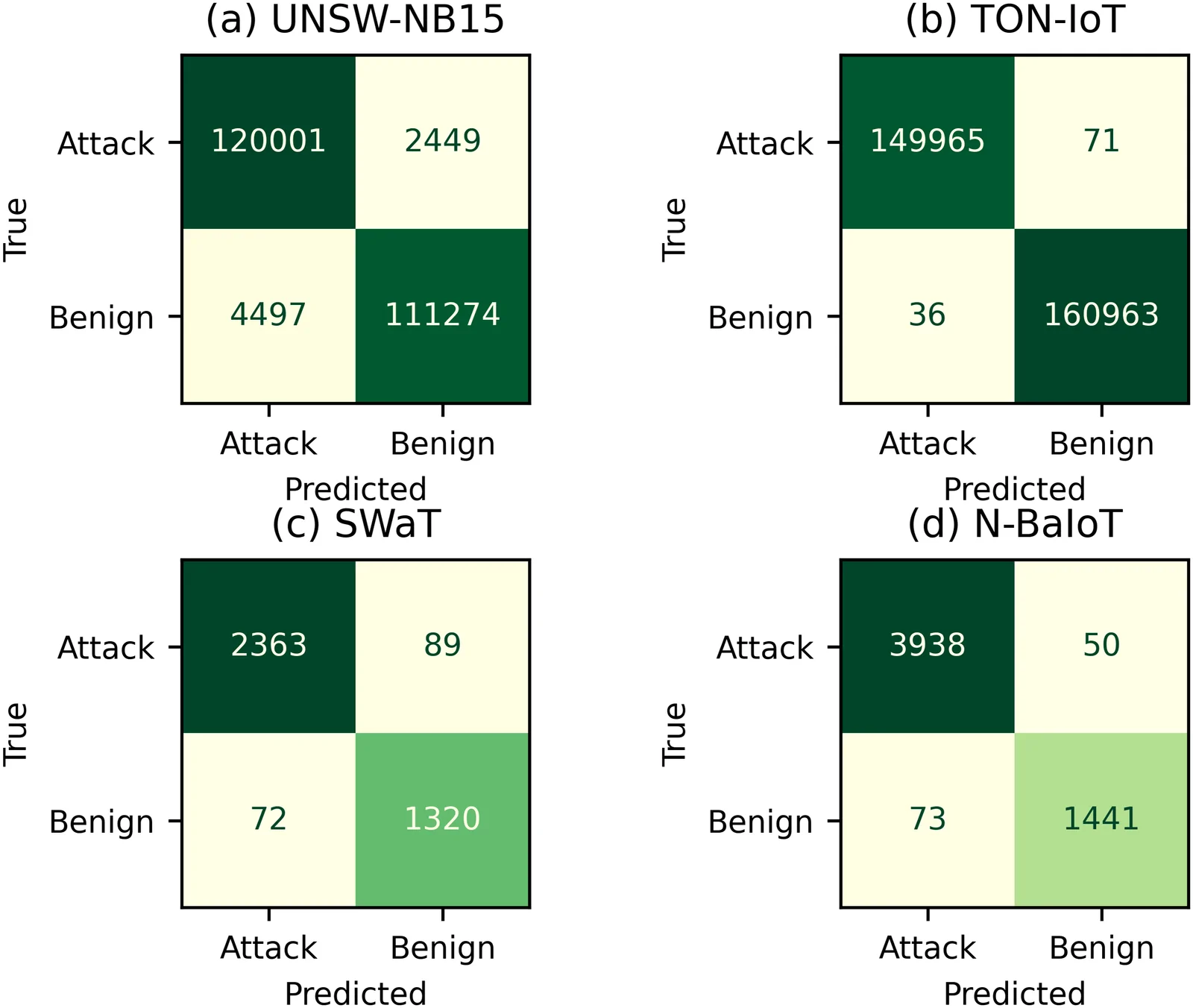
Binary confusion matrices of the TAG framework on the four CPS datasets.

**Fig 17:**
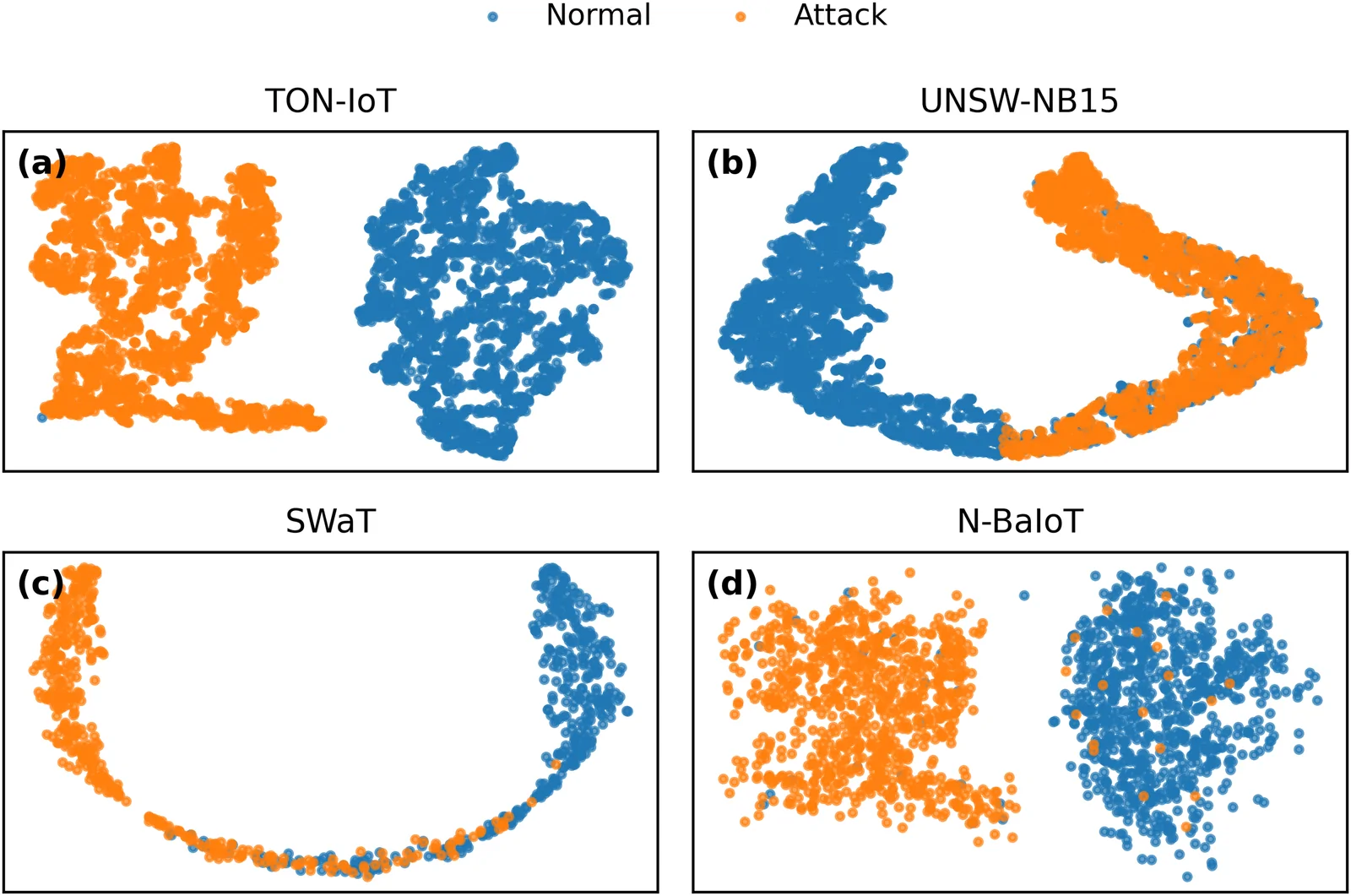
The t-SNE visualization of the latent feature representations learned by the proposed TAG framework for the datasets- a) TON-IoT, b) UNSW-NB15, c) SWaT, and d) N-BaIoT.

Uncertainty quantification is incorporated into the pipeline by empolying Monte Carlo Dropout (MC-Dropout) during inferencing. With the MC-Dropout, multiple stochastic forward passes are performed keeping the dropout active while testing the model. The average Bayesian confidence levels is presented in Fig 18. Excluding a few predictions at the uncertain zone; most of the prediction lies near 1 (between 0.7 and 0.9). This shows that the likelihood of overconfident predictions for unseen attacks is minimal, strengthening the claim that the TAG is well-calibrated in uncertain scenarios.

**Fig 18:**
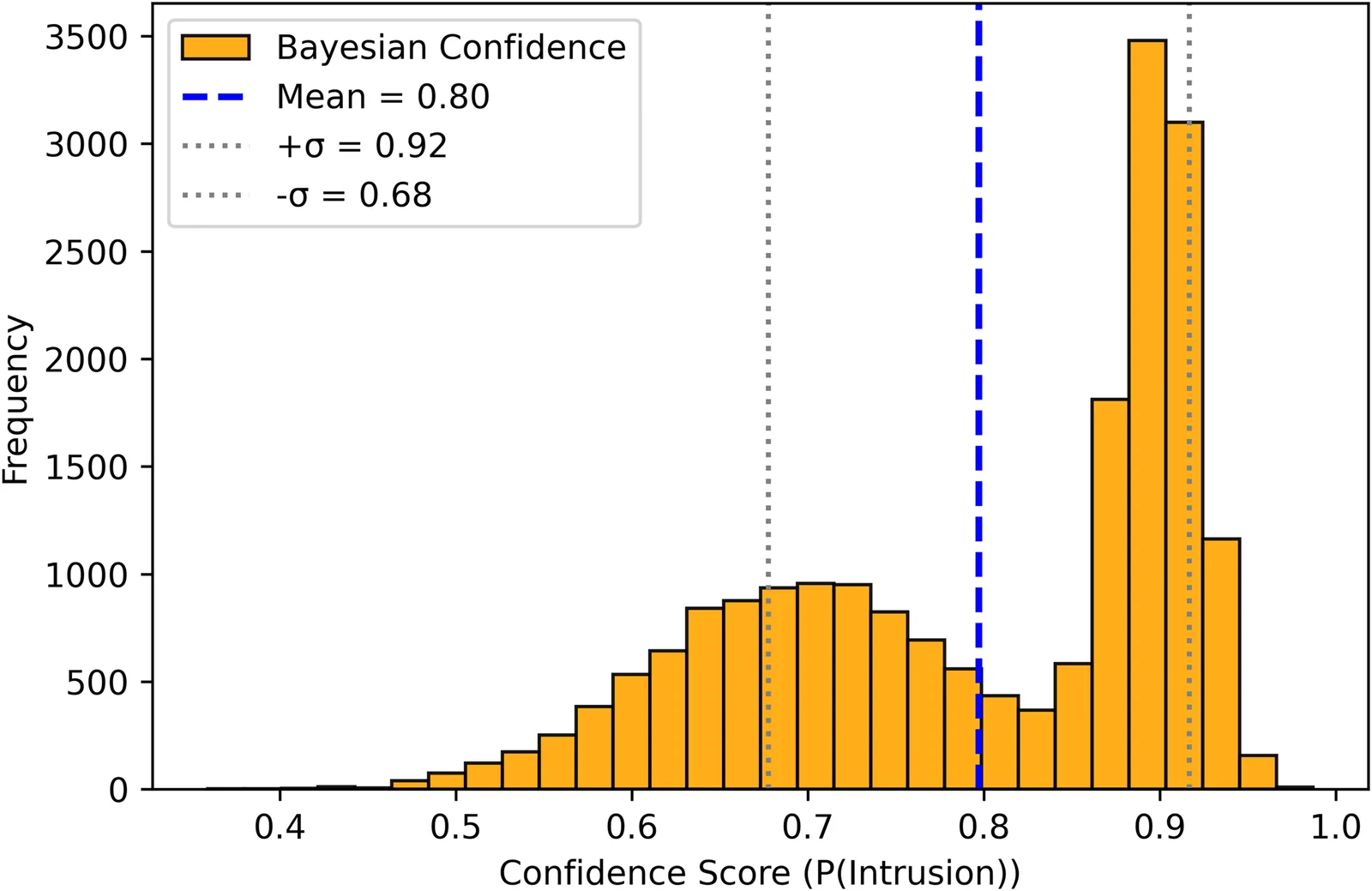
The Bayesian confidence scores with estimated mean obtained from the ensemble predictions.

Analyzing robustness, the proposed framework demonstrates consistentency across the CPS datasets despite differences in feature dimensionality: 42–51 features, dataset scale: 4,000–211,043 instances, and class distribution. TAG achieves high predictive performance, with ROC-AUC ranging from 0.982 to 1.000, F1-scores exceeding 0.91 across all datasets, and low standard deviations over the experimental runs. Notably, a stable performance is maintained on both large-scale datasets (TON-IoT containing over 211k samples) and small datasets (SWaT and N-BaIoT with 4k samples). The outcomes indicate that the proposed spatio-temporal architecture generalizes effectively without necessitating dataset-specific architectural modifications.

### Resource and complexity analysis

In a separate evaluation session, assessment of the resource utilization and computational cost is performed on the basis of the key metrics: training time, memory consumption and inference latency. Under the same resources setup, the system is evaluated in three different exclusive modes: 1) Swin transformer 2) STGNN and 3) Hybrid. Training time per epoch is measured in seconds (s), memory consumption in megabytes (MB), and inference latency in milliseconds (ms) during testing. Due to fusion and MC-Dropout layer, a moderate increase in training time and memory overhead was incurred, inference latency remained within the real-time constraints. The computational characteristics of TAG is validated by complexity analysis. The Swin transformer component operating on *N* tokens with M window size yields a complexity of $O(N\cdot M^{2})$. This shows a scalable global attention. The STGNN module exhibits a complexity of O(T·|E|·d), where T corresponds to temporal steps, |*E*| is the number of edges, and d the feature dimensionality. The Bayesian mechanism, introduces a linear overhead of *O*(*K*) during inference for *K* stochastic forward passes. As all components exhibit almost linear scaling behavior with respect to input size, applicability of the framework in large-scale CPS environments is supported. The analysis results are demonstrated in Table 7.

**Table 7:** Details about resource utilization and computational complexity of TAG.

Model	Training Time (s)	Memory Usage (MB)	Inference Latency (ms)	Time Complexity
Temporal Transformer	48.7	1850	82	*O*(*NM*^2^)
STGNN	58.9	2240	95	*O*(*T*|*E*|*d*)
Cross-Attention Fusion	11.3	420	17	*O*(*F*^2^)
Uncertainty Calibration	4.8	190	6	*O*(*F*)
TAG (Proposed)	67.5	2165	108	*O*(*NM*^2^ + *T*|*E*|*d* + *F*^2^ + *F*)

The table summarizes the computational characteristics of the major components of the proposed TAG framework. The transformer exhibits quadratic complexity with respect to the size of temporal window. The STGNN component scales linearly with graph connectivity and effectively captures spatial dependencies among CPS entities. The cross-attention fusion module introduces a moderate computational overhead for integrating temporal and graph representations, whereas the uncertainty calibration component contributes only a negligible increase in memory and runtime requirements. Collectively, the computational analysis indicates that TAG maintains favorable scalability characteristics despite integrating multiple advanced learning modules. The computational cost of TAG is observed from four key components- Transformer, STGNN, fusion, and MC-Dropout. Swin Transformer introduces multi-head self-attention mainly because of temporal sequence length, embedding dimension, and local window size. STGNN works on graph-based message passing where the cost depends on the number of nodes, and connectivity. The cross-attention fusion module introduces additional interaction between the temporal and spatial representations. Finally, as Bayesian MC-Dropout processes multiple stochastic forward passes, the inference-time overhead is increased. The components, though increase computational requirements, however, each contributes a distinct capability to the overall performance of the framework.

For the resource and complexity analysis, data about standard methods are taken from the literature, see Table 8. However, as runtime and memory statistics are not reported in the research papers, missing values as represented by “N/A” for fairness. The results endorsed favorable computational footprint of the proposed system.

**Table 8:** Resource utilization of representative intrusion detection systems.

Method	Accuracy	Throughput (attacks/s)	Inference Latency (ms)
Federated GNN	0.99	N/A	N/A
VGAE	0.97	N/A	N/A
CPS-GUARD	0.98	N/A	N/A
Continuum	0.98	N/A	N/A
TAG (Proposed)	0.98	9.7	103

The table compares the resource utilization of representative intrusion detection approaches in terms of classification accuracy, processing throughput, and inference latency. “N/A” indicates that the corresponding performance metric was not reported in the original study.

### Ablation study

To thoroughly evaluate significance of each component of the TAG framework, a comprehensive ablation study is conducted. The intent was to quantify the impact of various modules of the framework on the overall performance of TAG. Average of the accuracies- 0.99, 0.98, 0.99, 0.97, of the state-of-the-art methods- Continuum [43], CPS-GUARD [47], Federated attention-based GNN [48], and Multi-stage VGAE [46] is taken as the baseline. Starting from the full configuration of TAG, different ablated variants are assessed by systematically removing a component while keeping all other settings unchanged. The following configurations are examined in this regard:

(i) **Without Temporal Modeling (No Temporal):** The temporal transformer module was excluded and the model was set to rely on graph-based representations.
(ii) **Without Graph Learning (No Graph):** The graph representation module was removed where detection was performed on the basis of temporal features only.
(iii) **Without Cross-Attention Fusion:** The integration (through cross-attention mechanism) of graph and temporal representations was detached.
(iv) **Without Uncertainty Calibration:** The uncertainty-aware module was disabled to assess the influence of confidence estimation.
(v) **Graph-Only and Temporal-Only Variants:** Additional experiments were performed where only the graph or temporal modules where kept active to assess the standalone effectiveness of the modules.

As demonstrated in Fig 19, and Table 9 removing the adaptive graph learning module, the F1-score dropped substantially to 81%, indicating significance of the graph-based modeling. Similarly, the temporal module is also important because without the module accuracy drops to 85%. In contrast, removing the cross-attention mechanism and uncertainty calibration resulted in only marginal performance reductions, yield accuracy of 94% and 92.8%, respectively. The Graph-Only setup achieved 94.8%, while the Temporal-Only variant attained 92.64%. These outcomes indicate that, each representation independently captures substantial meaningful intrusion characteristics.

**Fig 19:**
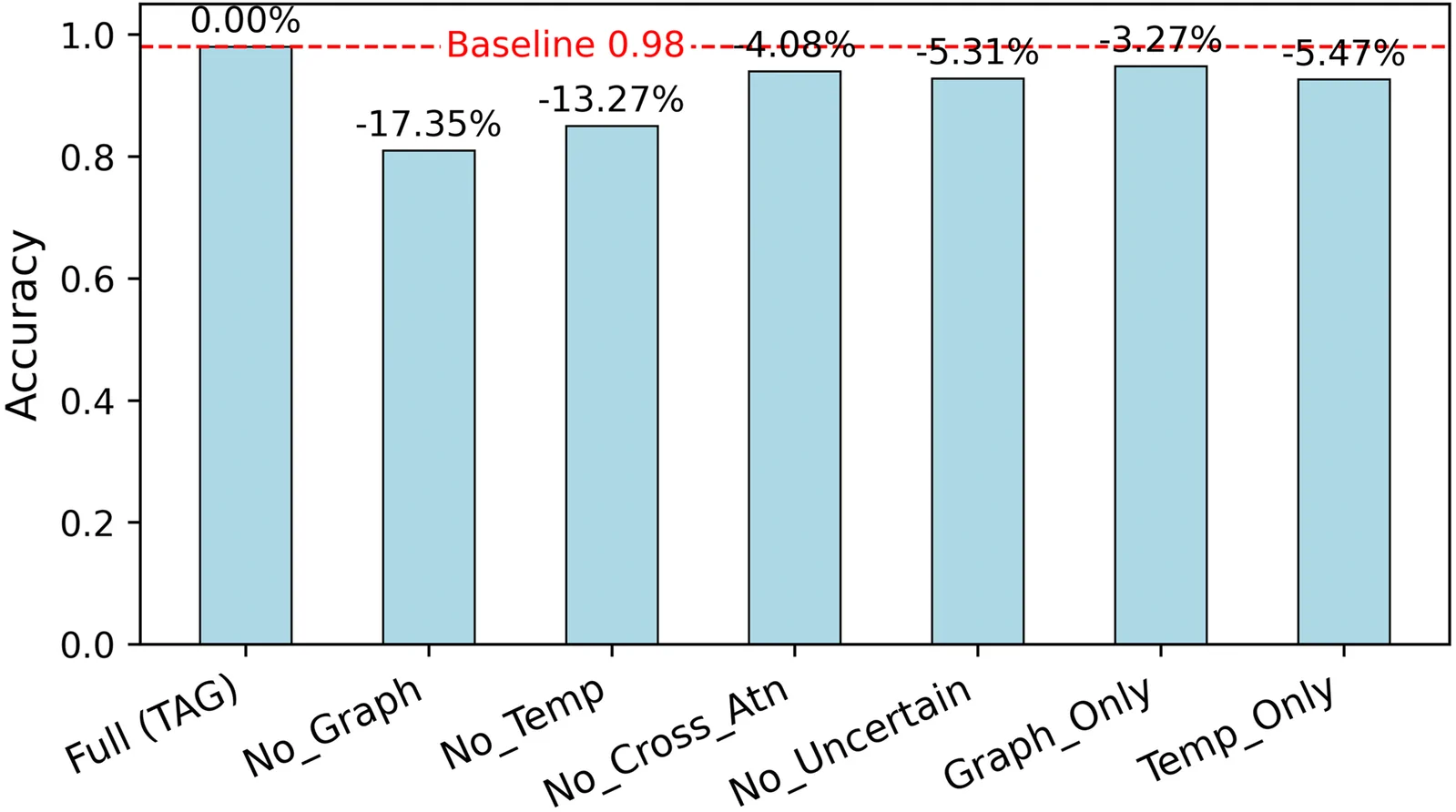
Outcomes of the ablation study.

**Table 9:** Ablation analysis of the proposed TAG framework.

Configuration	Accuracy (%)	F1-score (%)	Relative Change(%)
Full TAG	98	98	–
Without Temporal Modeling	85	83.6	−13.0
Without Graph Learning	80.1	81	−17.35
Without Cross- Attention Fusion	94.0	93.7	−4.0
Without Uncertainty Calibration	92.8	91.9	−5.2
Graph-Only	94.8	94.4	−3.2
Temporal-Only	92.64	92.1	−5.36

Additional experiments revealed nominal drop in accuracy for the Graph-Only and Temporal-Only configurations, suggesting that each representation independently captures meaningful intrusion characteristics; however, neither configuration achieves the performance attained by the complete framework. This afirms that temporal dynamics and graph topology provide complementary information for the cross-attention mechanism. The findings also validate the architectural design adopted in TAG and highlight need of the joint modeling approach of TAG for the security of complex CPS environment.

### Comparative analysis

After showcasing the results obtained, performance of the system is compared with some state-of-the-art IDs. Comparative analysis is performed from various perspectives like precision, recall, F1 score and time lapse in detecting an intrusion attack. In the analysis, statistical data about the other systems are taken from the literature: [43,46–52] As shown in Table 10, performance of the proposed framework is comparable to the contemporary IDS approaches with competitive precision, recall, and F1-score across the heterogeneous CPS datasets. In addition, TAG exhibits favorable inference characteristics, making it suitable for practical deployment in real-time CPS monitoring environments. The relative improvement or degradation is computed with respect to the average performance of four baseline methods: Continuum, CPS-GUARD, Federated GNN, and Multi-stage VGAE. However, the marginal misclassification is because of the class imbalance and variations in attack characteristics. Retraining with oversampling and by applying the weighted loss technique evidently enhanced performance of the system. Besides accuracy, time taken in detecting an intrusion is crucial to anticipate applicability of an IDS in real-world scenarios. Comparative analysis in terms of real-time detection of intrusion attacks is performed with the well-known traditional and DL based systems. For the experimentation, 50 flows per class were selected from each dataset and the average latency is compared with that of the baseline IDS. Corresponding to an average inference latency of 103 ms per sample, the framework is capable to trace 9.7 intrusion instances per second. The observed efficiency can be attributed to the effective integration of graph learning, temporal representation modeling, and attention-guided feature fusion enabling rapid inference without compromising accuracy.

**Table 10:** Comparative analysis of the proposed TAG framework with the standard IDS methods.

Category	Method	Precision	Recall	F1-Score	Relative Difference
Traditional	KNN	0.96	0.96	0.96	2.04% ↓
	RF	0.98	0.97	0.97	1.02% ↓
	ID3	0.98	0.97	0.98	0.00%
	AdaBoost	0.77	0.84	0.77	11.43% ↓
	Naïve Bayes	0.88	0.88	0.84	14.29% ↓
	SVM	0.68	0.66	0.67	31.63% ↓
	DT	0.79	0.80	0.79	19.39% ↓
ML/DL	Continuum	1.00	0.96	0.98	Comparable
	DT	0.79	0.80	0.79	19.39% ↓
	GBT	0.85	0.86	0.86	12.24% ↓
	2D-CNN	0.85	0.85	0.88	10.20% ↓
	STIDM	0.99	0.98	0.99	1.02% ↑
	NDMR	0.86	0.88	0.86	12.24% ↓
	Federated GNN	1.00	0.99	0.99	0.01% ↑
	Multi-stage VGAE	0.98	0.98	0.97	0.01% ↓
	CPS-GUARD	0.98	0.99	0.98	Comparable
	MLP	0.89	0.87	0.87	11.22% ↓
	L-SVM	0.84	0.76	0.79	19.39% ↓
	QDA	0.78	0.77	0.77	21.43% ↓
	SparseAE + SVM	0.96	0.76	0.86	12.24% ↓
	E-GraphSAGE	0.91	0.94	0.79	19.39% ↓
	PPT-GNN	0.98	0.96	0.93	5.10% ↓
	Op-ReDMAT	0.97	0.98	0.97	0.20% ↓
	GWO-CNN	0.96	0.97	0.97	0.94% ↓
	MHA-Transformer	0.91	0.92	0.92	6.12% ↓
	**TAG (Proposed)**	**0.97**	**0.96**	**0.98**	**Comparable**

### Reliability and significance analysis

Statistical reliability of the TAG system is systematically assessed through ten independent runs with different initialization seeds. The complementary aspects of Confidence Intervals (CI), and Shapiro-Wilk testing [53] are observed while testing the significance with t-test and Wilcoxon test. The CI is used to quantify the metrics uncertainty, whereas the Shapiro-Wilk is utilized to examine normality of the distribution obtained. Though the significance level- α of 0.05 is used however different CI values are obtained for each metric because of the different standard deviation;


$$\begin{array}{c} CI=\bar{x}\pm t_{\alpha /2,\;n-1}\;\frac{s}{\sqrt{n}} \end{array}$$ (36)

Initially, normality testing with Shapiro–Wilk is used to ensure which test is to be performed. The paired t-test is applied if the null hypothesis of normality is satisfied (*p* > 0.05); otherwise, the Wilcoxon signed-rank test is utilized. In terms of accuracy, our method (0.98±0.010) is statistically comparable to Continuum and CPS-GUARD, though slightly lower than the Federated GNN (0.99) is reported. Similarly, in terms of precision, our method (0.970±0.011) is equivalent to Multi-stage VGAE and CPS-GUARD. As shown in Fig 20 and Table 11, outcomes of the proposed method is comparable to Multi-stage VGAE and attains F1 score (0.98±0.008) equivalent to the CPS-GUARD. Overall, the outcomes demonstrate comparability of TAG with precision and recall thereby validating its effectiveness in CPS environments.

**Fig 20:**
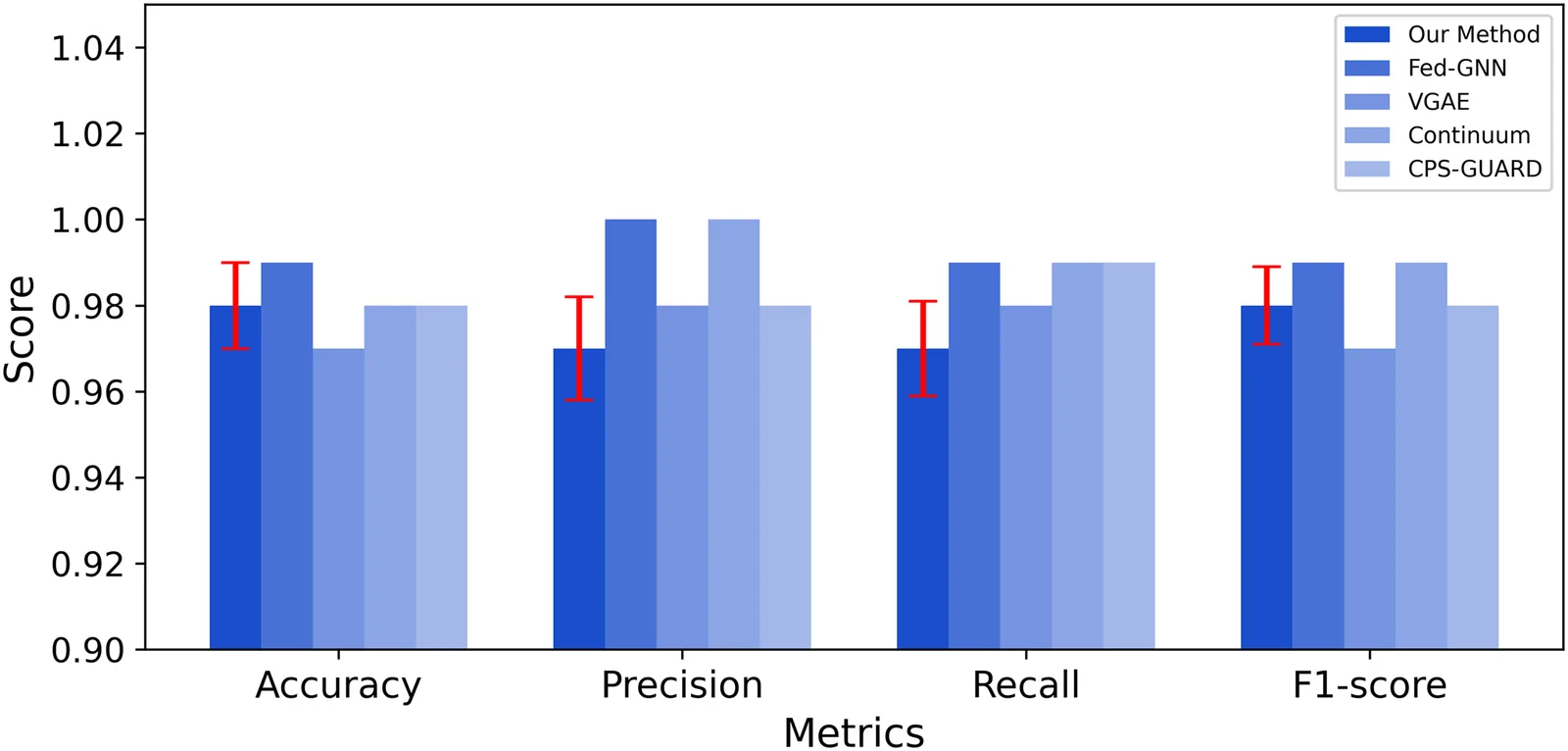
Comparison of the standard methods with error bars and CI (of 10 runs).

**Table 11:** Results of the reliability and statistical significance assessment.

Metric	TAG (μ±CI)	Fed. GNN	MS VGAE	Cont.	CPS -GUARD	Test	*p*-value	Significance
Accuracy	0.980 ±0.025	0.99	0.97	0.98	0.98	t-test	0.021–0.045	Comparable to all; slightly lower than Fed-GNN
Precision	0.970 ±0.011	1.00	0.98	1.00	0.98	t-test	0.038–0.041	Comparable to all except Fed-GNN and Continuum
Recall	0.960 ±0.014	0.99	0.98	0.99	0.99	Wilcoxon	0.027–0.052	Slightly lower than Fed-GNN, Continuum and CPS-GUARD
F1-score	0.980 ±0.008	0.99	0.97	0.99	0.98	t-test	0.039–0.046	Equivalent to Fed-GNN and Continuum; higher than VGAE and CPS-GUARD

## Conclusion and future work

Cyber-Physical Systems (CPSs) is gaining prominence in smart infrastructure, industrial automation, and pervasive networks. Though, Graph Neural Networks (GNNs) have been extensively utizlied for context-aware security applications, such frameworks mainly rely on snapshot-based graph representations. Overlooking temporal dependencies, time-dependent and multi-stage attacks, such as stealthy intrusions and slow-probing evade detection. To overcome these challenges, a hybrid framework is proposed in this study to model spatio-temporal dependencies by intermingling Swin Transformer with STGNN. Employing post-hoc Bayesian inference by leveraging MC-Dropout, predictive uncertainty is evaluated to enhance reliability and interpretability in decision. Morevoer, shifted-window transformer and dual-path graph aggregations are used to efficiently capture structural variations along-with temporal changes in CPS operations. The proposed unified framework exhibits effortless applicability through its multi-level intrusion detection mechanism across diverse benchmark datasets. As our future work, the framework will be augmented to address data imbalance by incorporating unsupervised and weakly supervised learning models. Besides, the computational efficiency will be improved for the deployment in large-scale real-time CPS environments.

## Supporting information

S1 Appendix Supplementary mathematical notation and methodological details of the TAG framework are provided in S1 Appendix. (TEX)
